# Targeting non-coding RNAs with the CRISPR/Cas9 system in human cell lines

**DOI:** 10.1093/nar/gku1198

**Published:** 2014-11-20

**Authors:** Tsui-Ting Ho, Nanjiang Zhou, Jianguo Huang, Pratirodh Koirala, Min Xu, Roland Fung, Fangting Wu, Yin-Yuan Mo

**Affiliations:** 1 Department of Pharmacology/Toxicology and Cancer Institute, University of Mississippi Medical Center, Jackson, MS 39216, USA; 2 Department of Gastroenterology, Affiliated Hospital of Jiangsu University, Zhenjiang, Jiangsu 212001, China; 3 System Biosciences, Mountain View, CA 94043, USA

## Abstract

The CRISPR/Cas has been recently shown to be a powerful genome-editing tool in a variety of organisms. However, these studies are mainly focused on protein-coding genes. The present study aims to determine whether this technology can be applied to non-coding genes. One of the challenges for knockout of non-coding genes is that a small deletion or insertion generated by the standard CRISPR/Cas system may not necessarily lead to functional loss of a given non-coding gene because of lacking an open reading frame, especially in polyploidy human cell lines. To overcome this challenge, we adopt a selection system that allows for marker genes to integrate into the genome through homologous recombination (HR). Moreover, we construct a dual guide RNA vector that can make two cuts simultaneously at designated sites such that a large fragment can be deleted. With these approaches, we are able to successfully generate knockouts for miR-21, miR-29a, lncRNA-21A, UCA1 and AK023948 in various human cell lines. Finally, we show that the HR-mediated targeting efficiency can be further improved by suppression of the non-homologous end joining pathway. Together, these results demonstrate the feasibility of knockout for non-coding genes by the CRISPR/Cas system in human cell lines.

## INTRODUCTION

It is well known that the human genome is actively transcribed; however, there are only about 20, 000 protein-coding genes (1), accounting for about 2% of the genome, and the rest of the transcripts are non-coding RNAs including microRNAs and long non-coding RNAs (lncRNAs). For instance, over 56, 000 human lncRNAs have been identified to date (2); however, the biological function for most of them is not known. Therefore, there is a critical need for functional studies of lncRNAs. A most commonly used approach for gene functional study is knockdown by RNA inference (RNAi) which is mainly functional in the cytoplasm where RISC complexes are located (3). However, many lncRNAs are localized to the nucleus (4), which can make it difficult to achieve robust knockdown. Thus, genetic editing at the genomic level provides a better alternative because it targets the genomic DNA. There are several genetic tools available for this purpose, including zinc finger nuclease (ZFN) (5) and transcription activation-like element nuclease (TALEN) (6). For example, type II restriction enzyme FokI is often used as a cleavage domain in ZFN (7); similarly, engineered TAL effectors can also be fused to the cleavage domain of FokI to create TALENs for genome editing (8). Recently, a novel genetic engineering tool called clustered regularly interspaced short palindromic repeats (CRISPR)/CRISPR‐associated (Cas) system is more advanced because of easy generation and high efficiency of gene targeting (9). Importantly, it only requires changing the sequence of the guide RNA (gRNA); and it can be directly delivered into embryos, to generate sequence-modified animals (10–13). Furthermore, multiplexing capability of CRISPR/Cas makes it possible to target multiple genes simultaneously (12).

As a bacterial adaptive immune defense mechanism, the CRISPR/Cas9 system has been known for a long time. There are three types of CRISPR/Cas systems (14). Type II system has been well studied originally because it offered practical applications in the dairy industry to generate phage-resistant *Streptococcus thermophilus* strains (15). In addition, type II uses single effector enzyme such as Cas9 to cleave dsDNA. Because of this unique feature, CRISPR/Cas9 system has been extensively studied as a genetic engineering tool from bacteria to mammals.

Since the first report in the bacterial system (16), a large number of papers have been published on the utility of CRISPR/Cas system in various eukaryotic organisms including yeast, drosophila, zebrafish, mouse and human cells (11,13,17–22). In the first landmark paper, Jinek *et al.* demonstrated that the dual-tracrRNA:crRNA, which occurs in nature, can be engineered as a single RNA chimera to function as just like dual-tracrRNA:crRNA, and is able to direct sequence-specific Cas9 dsDNA cleavage (16). Mali *et al.* reported a knockout approach using human codon optimized Cas9 (hCas9) with custom gRNA (a chimeric form) in human cells (21). Using the endogenous AAVS1 locus as an example, they demonstrated that targeting rates reach from 2 to 25% depending on cell types used. A similar study showed that Cas9 nucleases can be directed by short RNAs to induce precise cleavage at endogenous genomic loci in human and mouse cells (19).

However, all of these studies are focused on protein-coding genes. Information about targeting non-coding RNAs is scarce. Although two reports used CRISPR/Cas9 to knockout microRNA in the zebrafish genome (23) and to target a maternally expressed lncRNA, Rian, in mouse model (24), none of them used homologous recombination (HR)-based selection system or a dual gRNA approach with two gRNAs expressed in the same vector. Therefore, the present study aims to explore an applicability of CRISPR/Cas system through an HR-based selection and/or dual gRNA approach to non-coding genes in human cell lines.

## MATERIALS AND METHODS

### Reagents

Primary antibodies were obtained as follows: PDCD4 monoclonal from Sigma-Aldrich (St. Louis, MO, USA); GAPDH from ProteinTech (Chicago, IL, USA). Secondary antibodies conjugated with IRDye 800CW or IRDye 680 were purchased from LI-COR Biosciences (Lincoln, NE, USA). Polymerase chain reaction (PCR) primers were purchased from IDT (Coralville, IA, USA) and siRNAs from Dharmacon (Lafayette, CO, USA).

### Cell culture

HEK293, HEK293T, HCT-116, MCF-7 and LNCaP were obtained from ATCC (Manassas, VA, USA) and cultured in Dulbecco's modified Eagle's medium (DMEM) or RPMI-1640 with 10% fetal bovine serum (FBS) (Sigma). All media contained 2 mM glutamine, 100 units of penicillin/ml and 100 mg of streptomycin/ml. Cells were incubated at 37ºC and supplemented with 5% CO_2_ in a humidified chamber.

### Construction of plasmids

The high fidelity enzyme Phusion (NEB, Ipswich, MA, USA) was used to amplify respective DNA fragments by PCR to make these constructs. gRNA design was based on CRISPR design (http://crispr.mit.edu/) or CHOPCHOP (https://chopchop.rc.fas.harvard.edu/). For single gRNA cloning we purchased a set of self-complementary oligos from IDT and then ligated into PrecisonX^TM^ Cas9 SmartNuclease (Cat# CAS900A-1, System Biosciences, Mountain View, CA, USA) according to manufacturer's protocol. For dual gRNA cloning, we introduced dual gRNAs under H1 and U6 promoter along with gRNA scaffold downstream of hCas9 to generate a dual gRNA vector using multiplex gRNA cloning kit (Cat# CAS9-GRNA-KIT, SBI). To construct a donor vector for miR-21 or miR-29a, we used MCS1-LoxP-EF1a-GFP-T2A-Puro-P2A-hsvTK-pA-LoxP-MCS2 (Cat#: HR210PA-1, SBI). Left and right arms (∼800 bp each) were amplified from genomic DNA using primers miR-21-L-Spe-5.1; miR-21-L-Bsa1–3.1; and miR-21-R-Bbs-5.2 and miR-21-R-Sal-3.2 (Supplementary Table S1), and sequentially cloned into the vector by ColdFusion cloning kit (Cat# MC101B-1, SBI). To construct a donor vector for UCA1, lncRNA-21A or AK023948, we used a pSK backbone carrying LoxP-EF1a-GFP-T2A-Puro-pA-LoxP with restriction enzyme BamH1 on the left side and EcoR I on the right side. Left and right arms were then sequentially cloned into the vector using ColdFusion cloning kit. All PCR products were verified by DNA sequencing.

### Transfection of siRNAs/plasmid DNAs and selection

**Figure 1.**
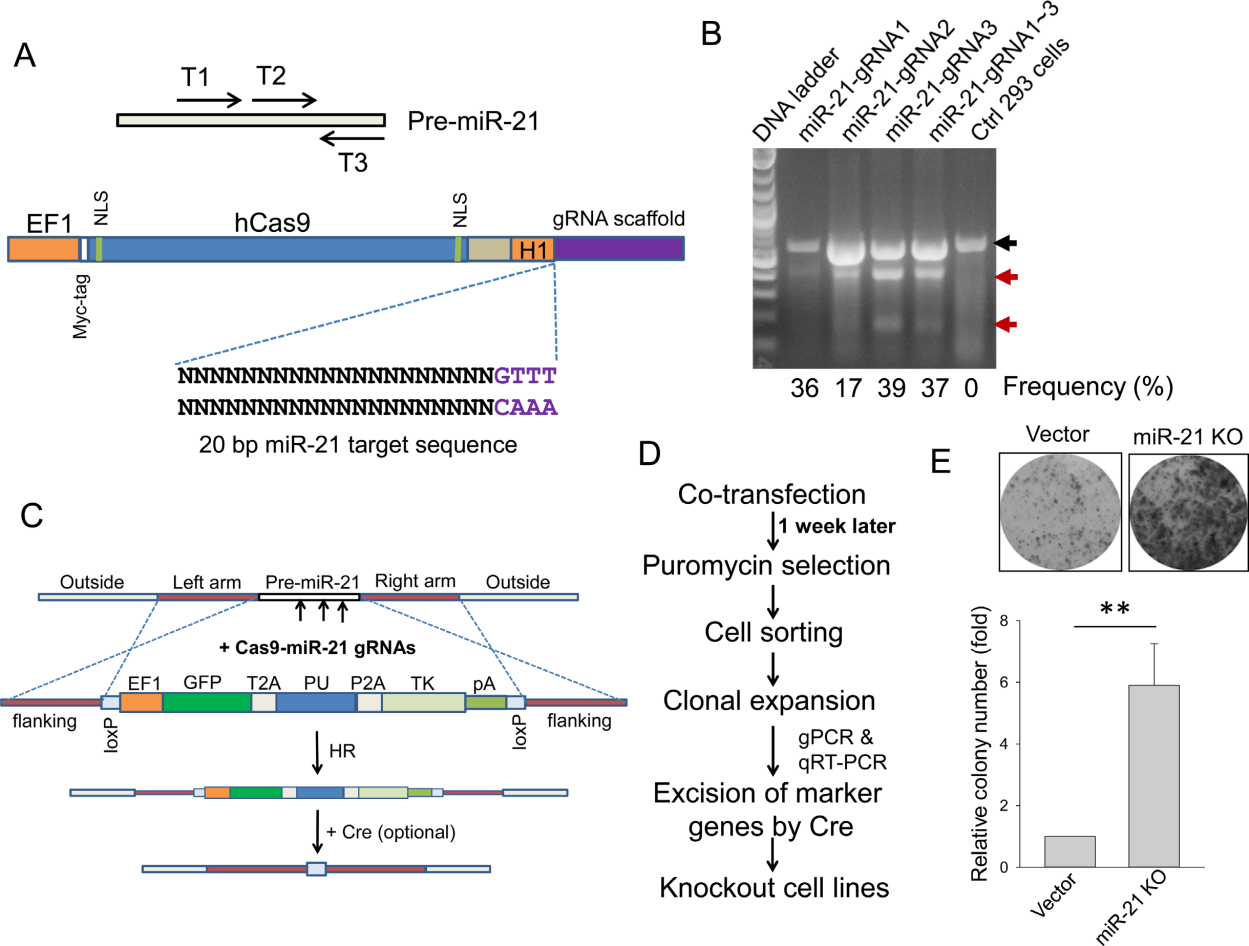
Knockout of miR-21 in HEK293 cells. (**A**) Strategy for making Cas9 all-in-one vector targeting miR-21 precursor. Three gRNA target regions are shown at the top panel; a single miR-21 gRNA each was cloned into the vector at the bottom panel as detailed in the Materials and Methods section. (**B**) Detection of indels in miR-21 knockout cells by T7 endonuclease 1 or Surveyor nuclease assay, as detailed in the Materials and Methods section. The black arrow indicates wild-type band and the red arrows indicate mutant bands, with relative cleavage frequency shown at the bottom. (**C**) A donor vector for targeting miR-21 through homologous recombination (HR). pA, poly A signal. (**D**) A procedure of selection and identification of knockout clones. (**E**) miR-21 knockout increased colony number, as determined by colony formation assay for a mixed pool after puromycin selection. Values are means of ± SE (*n* = 3). **P < 0.01.

Cells were transfected with siRNAs using RNAfectin reagent (Applied Biological Materials, Vancouver, Canada) following the manufacturer's protocol, as described previously (25). siRNA sequences have been described for Ku70 (26) and Lig4 and XRCC4 (27). The selection procedure was listed in Figure 1D. In brief, constructs carrying hCas9 and single gRNA or dual gRNAs, along with donor vector, were introduced into cells by transfection using DNAfectin (Applied Biological Materials). Next day, the cells were replaced with fresh medium, and re-seeded when the cells became confluent. One week later, the transfected cells were subject to puromycin selection at concentrations of 0.5, 1.0 or 1.5 μg/ml depending on the type of cells used; the purpose was to maximize recombination and minimize the random insertion. Two weeks after selection, surviving cells were sorted by fluorescence-activated cell sorting (FACS) based on GFP signal into individual wells of 96-well plates and incubated for 2–3 weeks. The sorted clones were then expanded in 12-well plates, followed by extraction of genomic DNA or total RNA for genomic PCR or quantitative reverse transcription (qRT)-PCR using standard methods. To detect HR events, we performed junction PCR using primers derived from outside sequence and the donor vector sequence, and then verified them by DNA sequencing of junction PCR products.

### RNA preparation and RT-PCR

For RT-PCR, we isolated total RNA using Direct-zol™ RNA MiniPrep (Zymo Research, Irvine, CA, USA) per the manufacturer's protocol and used 0.5 μg RNA to synthesize cDNA by RevertAid Reverse Transcriptase (ThermoFisher) with random primers. Finally, the resultant cDNA was used for PCR reactions. PCR annealing temperature varied depending on the primers used. To detect miR-21, we used the polymerase A method, followed by SYBR Green qPCR as described previously (28). RNU1 and 5s RNA were used as an internal control.

### Western blotting

Cells were harvested and protein was extracted from cells as previously described (25). The protein concentration was determined using a protein assay kit (Bio-Rad, Hercules, CA, USA) and samples were separated in sodium dodecyl sulphate polyacrylamide gels.

### Detection of mismatched duplexes by T7 endonuclease assay

HEK293 cells were transfected with hCas9 carrying miR-21 gRNAs as described above and then genomic DNA was isolated 72 h post transfection. Genomic region surrounding the target site for miR-21 was PCR amplified using Phusion high-fidelity DNA polymerase, and products were purified using Zymoclean™ Gel DNA Recovery Kit (Zymo Research, Irvine, CA, USA) following manufacturer's protocol. A total of 200 ng of the purified PCR products were mixed with 2 μl 10 X Buffer 2 (NEB) and ultrapure water to a final volume of 20 μl, and subject to a re-annealing process to enable heteroduplex formation: 95ºC for 10 min, 95–85ºC ramping at –2ºC/s, 85–25ºC at –0.25ºC/s and 25ºC for 1 min. After re-annealing, products were treated with 0.5-μl T7 endonuclease I (NEB) at 37ºC for 15 min and analyzed on 1.5–2% agarose gels.

### Excision of loxP by Cre

Cre mRNA was synthesized using mRNAExpress mRNA Synthesis Kit (Cat# MR-KIT-1, SBI) according to user manual's protocol. In brief, the Cre coding region was cloned into pMRNA vector. Then T7 promoter carrying Cre fragment was PCR amplified using primers the kit provided. Later, purified PCR product was used as a template to synthesis Cre mRNA. Cells were seeded at ∼20% and grown overnight, and then transfected with 0.5–1 μg Cre mRNA per well in a 12-well plate. Three days later the transfected cells were transferred to large dishes (10 cm), and individual colonies were formed within 2 weeks. The excision efficiency is dependent on transfection efficiency. Based on our experience, about 50–60% colonies had lost the marker genes, revealing non-green and puromycin sensitive.

### Statistical analysis

Comparisons between groups were analyzed using the Student's *t*-test (two groups) or a one-way analysis of variance (ANOVA) followed by *post hoc* Tukey test (multiple groups). Differences with *P* values less than 0.05 are considered significant.

## RESULTS

Knockout of protein-coding genes is relatively easy because a small deletion or insertion can disrupt the open reading frame so that no functional gene product is produced. However, non-coding genes are very different. Especially for lncRNAs, a small deletion or insertion may not necessarily cause functional knockout. To test the feasibility of non-coding gene knockout with CRISPR/Cas9 system, we chose two microRNAs, i.e. miR-21 (28) and miR-29a (29), and three lncRNAs, i.e. UCA1 (30), lncRNA-21A (31) and AK023948 (32) and tested them in various human cell lines. For miR-21, we adopted an all-in-one system, i.e. hCas9 and a single gRNA in the same vector. Thus, three individual gRNAs were designed to target pre-miR-21 (Figure 1A, top) and each of them was separately cloned into the vector (Figure 1A, bottom). After introducing a single gRNA or mixed three gRNAs into HEK293 cells by transfection, we performed T7 endonuclease assay and found that each of the three individual gRNAs or combination produced mismatched bands with targeting frequency of 17–39% (Figure 1B), suggesting that this approach is robust and miR-21 sequence is altered. However, we encountered a difficulty in identifying complete knockout clones. There was no complete miR-21 knockout among over 100 individual colonies screened. Possible reasons for this are: (i) if transfection efficiency is not 100%, those non-transfected cells will increase the screening effort; (ii) many established cell lines are polyploid. For example, HEK293 cells are hypotriploid. In this case, the cut may have to be simultaneously made on three chromosomes in order to get a complete knockout.

To facilitate the selection of complete knockout clones, we generated a donor vector in such a way that target fragment is replaced by marker genes (GFP and PU, the puromycin resistance gene, and TK, thymidine kinase gene) once it is integrated into the genomic DNA by HR (Figure 1C). GFP enables visualization of the transfected cells and cell sorting, and puromycin resistance gene allows selection of positive stable transfectants carrying targeted sequences by HR. TK is for selection of positive clones after the marker genes are excised at loxP sites by Cre when it is needed; it also helps to eliminate some remaining clones with random integration. Outside the markers, we introduced an ∼800-bp DNA fragment from targeting sites (left arm and right arm) for HR (Figure 1C). These vectors were simultaneously introduced into cells by transfection and then subject to puromycin selection as outlined in Figure 1D. To reduce the possibility that the donor sequence randomly integrate into the genome, we did not start puromycin selection until 1 week after transfection. We first tested targeting efficiency by counting surviving colonies in the presence of puromycin (1 μg/ml). Co-transfection of miR-21 gRNAs and donor vectors in HEK293 cells resulted in an ∼6-fold increase of colony number compared to vector control cells (Figure 1E).

**Figure 2.**
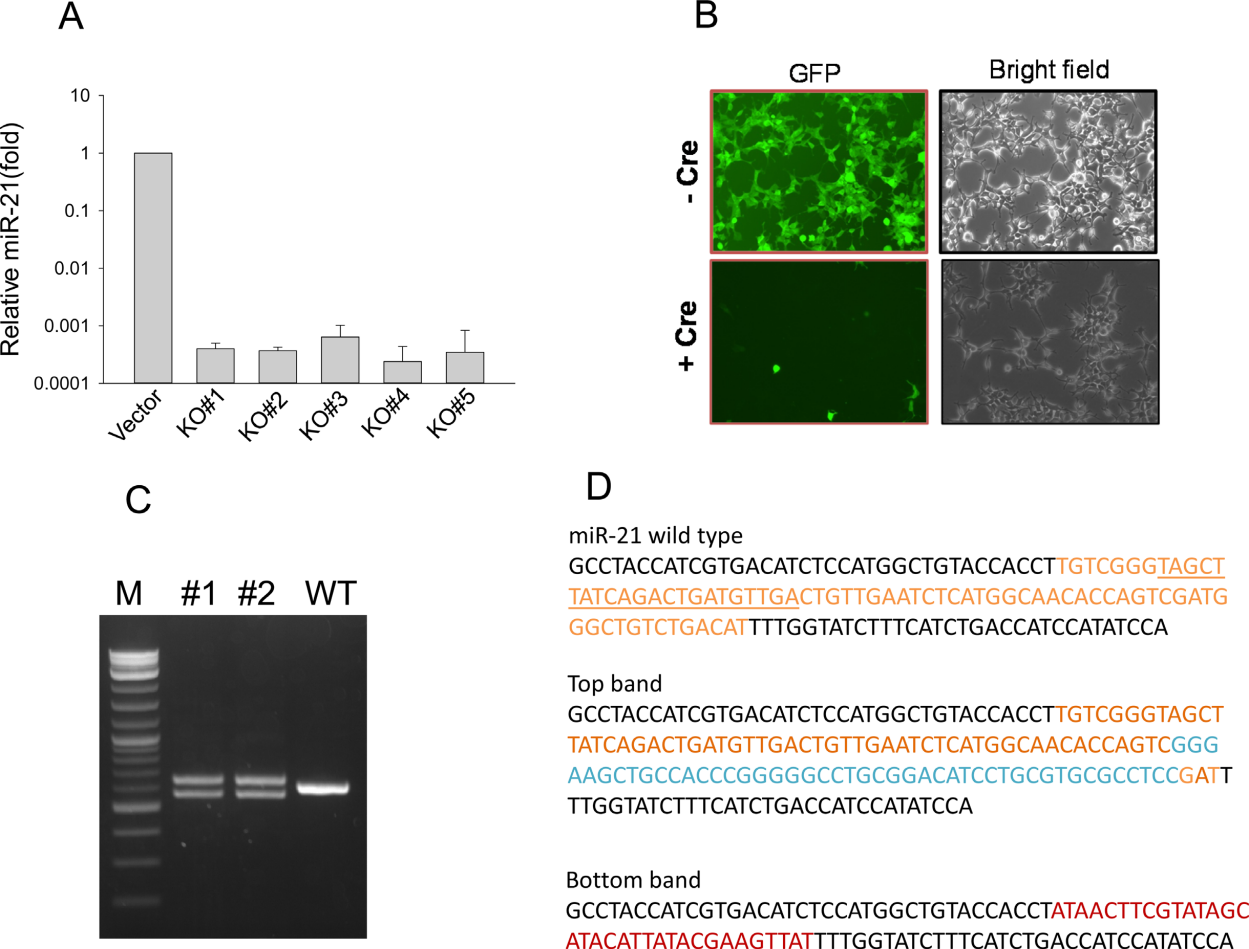
Identification of miR-21 KO clones and elimination of marker genes. (**A**) Detection of miR-21 level by qRT-PCR. Over a 1000-fold reduction was detected in all of the five miR-21 KO clones with *P* value <0.01 for all five clones. (**B**) Excision of marker genes in miR-21 KO clone #1 cells by Cre. Shown here are representative images. (**C**) Both miR-21 KO clones #1 and #2 carry distinct PCR products using primers miR-21–5.1 and miR-21–3.1. (**D**) DNA sequences for each of the PCR products from clones #1 and #2. Both clones revealed identical sequences. Insertion sequences are shown in blue and loxP sequences are in red.

These surviving cells were further subject to cell sorting by FACS based on GFP signal into 96-well plates, followed by expansion in 12-well plates. We recovered 10 individual clones from a 96-well plate. qRT-PCR analysis revealed that five clones were complete miR-21 knockouts because over a 1000-fold reduction in miR-21 level was detected for each of them (Figure 2A). Since the donor vector carries two loxP sites, we introduced Cre mRNA into each of them. As expected, excision of the marker genes generated non-green cells (Figure 2B). To determine the nature of sequence alterations for these five clones, we designed primers miR-21–5.1 and miR-21–3.1 which would produce an amplicon size of 614 bp for wild type (WT). As shown in Figure 2C, clones #1 and #2 each generated two bands, whereas the parental cells produced a single band (WT). DNA sequencing showed that these two clones were duplicates because the sequences for each of the two bands were exactly the same for clones #1 and #2. The top band carried a 14-bp deletion (ATGGGCTGTCTGAC) along with a 46-bp insertion, leading to a 32-bp net insertion; bottom band carried a 72-bp deletion (pre-miR-21), and one copy of loxP site (34 bp) was retained (Figure 2D). For clones #3 and #4, we detected two different sequences (Supplementary Figure S1) in addition to the loxP site copy, whereas clone #5 carried an 88-bp insertion. These results are consistent with the triploidy nature of this cell line. While one copy was replaced by donor vector through HR (i.e. the loxP copy), the rest two copies (# 3 and #4) had a deletion or insertion, suggesting that these cells have a strong non-homologous end joining (NHEJ) activity. Although these clones are ‘heterozygous’ by nature, they are complete knockouts for miR-21. In addition to miR-21, we also tested miR-29a knockout in HEK293 cells with the same approach and we were also able to obtain complete miR-29a knockout clones (not shown).

**Figure 3.**
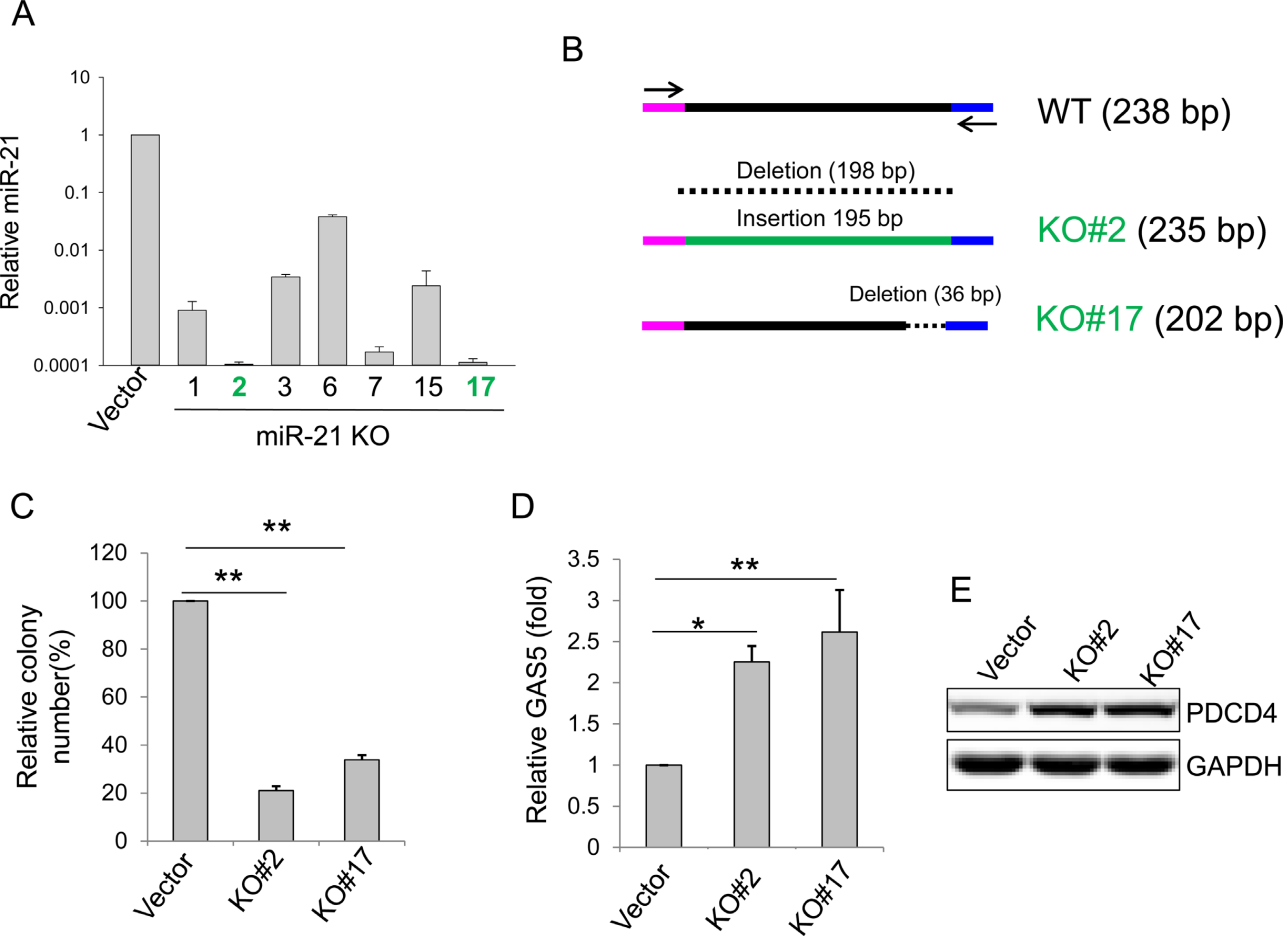
Characterization of miR-21 KO clones in HCT-116. (**A**) Detection of miR-21 in miR-21 KO clones by qRT-PCR; *P* value <0.01 for all seven clones compared to vector control. miR-21 KO #2 and #17 (in green) were selected for further analysis. (**B**) Detection of alterations at the target region of miR-21 KO #2 and #17 by DNA sequencing. (**C**) miR-21 knockout suppresses cell growth, as determined by colony formation assays. (**D**) miR-21 knockout increases the miR-21 target GAS5, as detected by qRT-PCR. (**E**) miR-21 knockout increases the miR-21 target PDCD4, as detected by western blot. Values in (C) and (D) are means of ± SE (*n* = 3). *P < 0.05 and **P < 0.01.

We then tested miR-21 knockout in colon cancer HCT-116 cells that are mainly diploid (www.atcc.org). miR-21 knockout caused over a 5-fold increase in colony number compared to vector control (Supplementary Figure S2A and B), which is comparable to the number of HEK293 knockout cells (Figure 1E). After cell sorting, the puromycin-resistant GFP-positive clones were picked out and expanded. Among over 20 clones, we identified seven clones with over 90% reduction of miR-21 by qRT-PCR (Figure 3A). Clones #2 and #17 were subject to further characterization. DNA sequencing of PCR products revealed that clone #2 carried a 198-bp deletion but with a 195-bp insertion; clone #17 carried a 36-bp deletion (Figure 3B; Supplementary Figure S2C). To determine the function of miR-21 knockout, we performed colony formation assays and found that cell growth of these clones was substantially slower than that of the control cells after 1-week incubation. Representative pictures of crystal violet-stained colonies are shown in Supplementary Figure S2D. The percentage of colony formation of miR-21 knockout clones decreased to 21.1%, and 33.9% in clones #2 and #17, respectively, compared to control (Figure 3C). Moreover, we found that two known miR-21 target genes, growth arrest-specific 5 (GAS5) (33) and programmed cell death 4 (PDCD4) (34), were significantly induced in the miR-21 knockout cells, as detected by qRT-PCR (Figure 3D) and western blot (Figure 3E), respectively, demonstrating that they are functional. Finally, to determine HR events, we performed junction PCR using primers miR-21-recom-5.2 and EF1-seq-3.2 (Supplementary Figure S3A) and found that while no PCR product was detected for donor + vector, HR rate was about 74% (14/19) for donor + miR-21 gRNAs (Supplementary Figure S2A). DNA sequencing of three randomly selected PCR products confirmed the HR event and all three products revealed identical sequences (Supplementary Figure S2B).

In addition, we also made miR-21 knockout in prostate cancer LNCaP and breast cancer MCF-7 cells. Knockout of miR-21 in LNCaP cells led to a 1.67-fold increase in colony number (Supplementary Figure S4A). Expression of miR-21 was decreased by 39.6% for a mixed pool, as detected by qRT-PCR (Supplementary Figure S4B). Similarly, knockout of miR-21 in MCF-7 cells resulted in a 2.77-fold increase in colony number (Supplementary Figure S4C) and miR-21 was decreased by 37.5% for a mixed pool (Supplementary Figure S4D).

**Figure 4.**
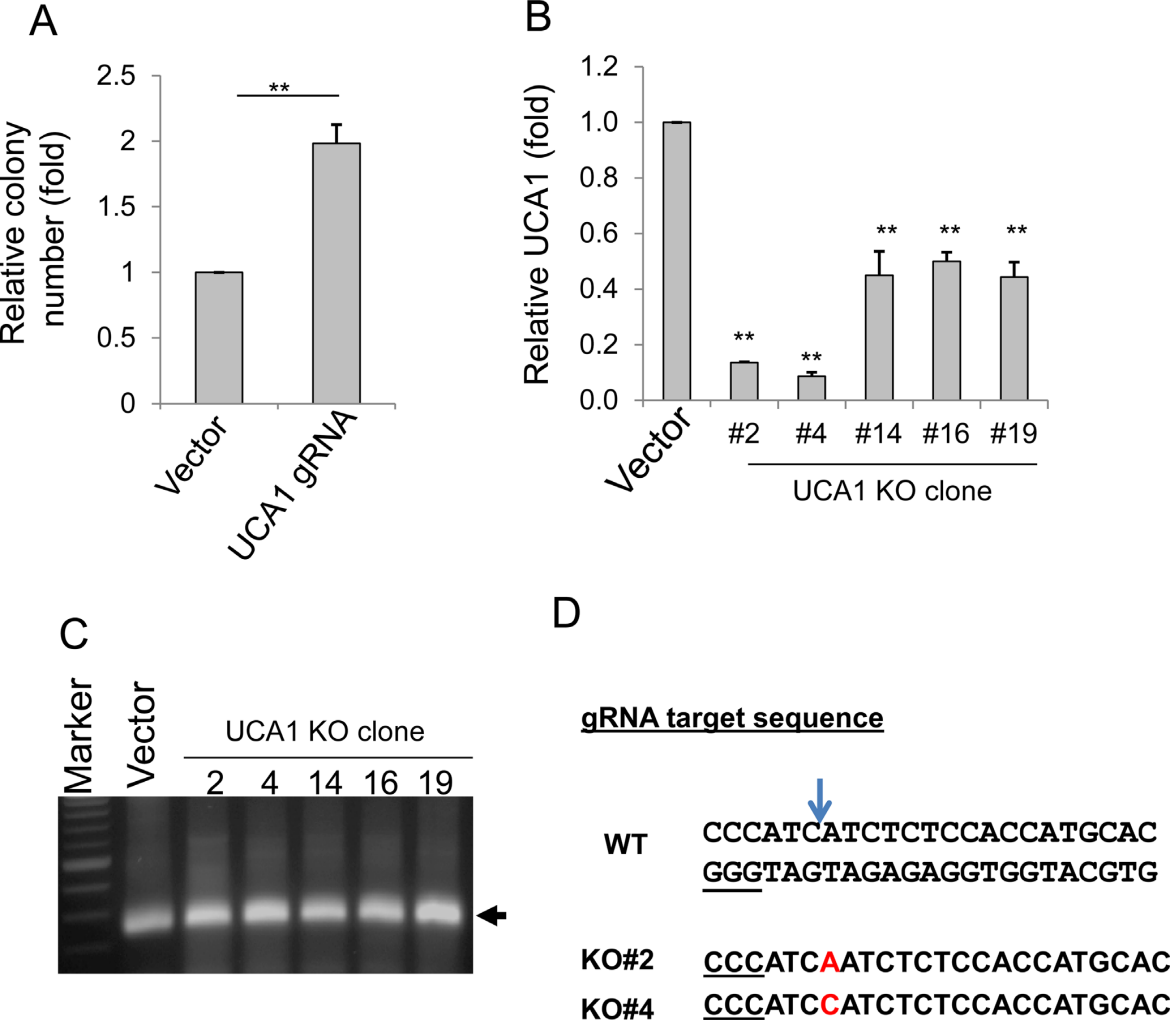
Generation of UCA1 knockout in HCT-116 cells. (**A**) Colony formation for vector and UCA1 KO for a mixed pool after puromycin selection. (**B**) Detection of UCA1 expression in UCA1 KO clones by qRT-PCR. (**C**) Detection of UCA1 target region in all five clones using genomic DNA as a template and primers UCA1-inside-5.3 and UCA1-inside-3.3. (**D**) Detection of a single nucleotide insertion (in red) in UCA1 KO#2 and #4, by DNA sequencing. The arrow indicates the cleavage site by Cas9. Underlined GGG is PAM sequence. Values in (A) and (D) are means of ± SE (*n* = 3). **P < 0.01.

Having demonstrated the successful knockout of miR-21, we then determined whether the CRISPR/Cas system can be applied to lncRNA genes, including UCA1, lncRNA-21A and AK023948. UCA1 has been shown to be a potential oncogene (30) and it contains three exons. We initially designed a single gRNA targeting UCA1 exon 1 (Supplementary Figure S5A). Colony formation assays with a mixed pool revealed that there was a 1.98-fold increase in colony number in UCA1 knockout compared to vector control (Figure 4A). Genomic PCR identified seven potential clones among 50 randomly picked single clones; qRT-PCR revealed that UCA1 level for clones #2 and #4 was reduced by about 90% while UCA1 level for clones #14, #16 and #19 was reduced by about 50% (Figure 4B). However, genomic PCR using primers derived from exon 1 region (UCA1-inside-5.3 and UCA1-inside-3.3) (Supplementary Figure S5B) revealed a positive band for all five clones (Figure 4C). DNA sequencing of this band identified a single nucleotide insertion in red (Figure 4D) for clones #2 and #4, respectively, whereas the band from clones #14, #16 and #19 was wild type, again, suggesting that NHEJ takes place in addition to HR.

These findings suggest that although at least one copy of donor sequence is integrated into genome by HR, not all cells have homozygous recombinants at UCA1 site. Instead, we were still able to detect the targeted UCA1 region by genomic PCR (Figure 4C), suggesting that the cells carry one copy of recombinant and one copy wild type or altered sequence at UCA1 site if it is diploid. It is evident that altered copy due to deletion or insertion may still produce UCA1, as suggested by qRT-PCR. Since microRNA is relatively short, a small deletion can cause disruption of mature microRNA sequence. However, for lncRNAs, such small deletions or insertions may not be sufficient to completely disrupt their expression.

**Figure 5.**
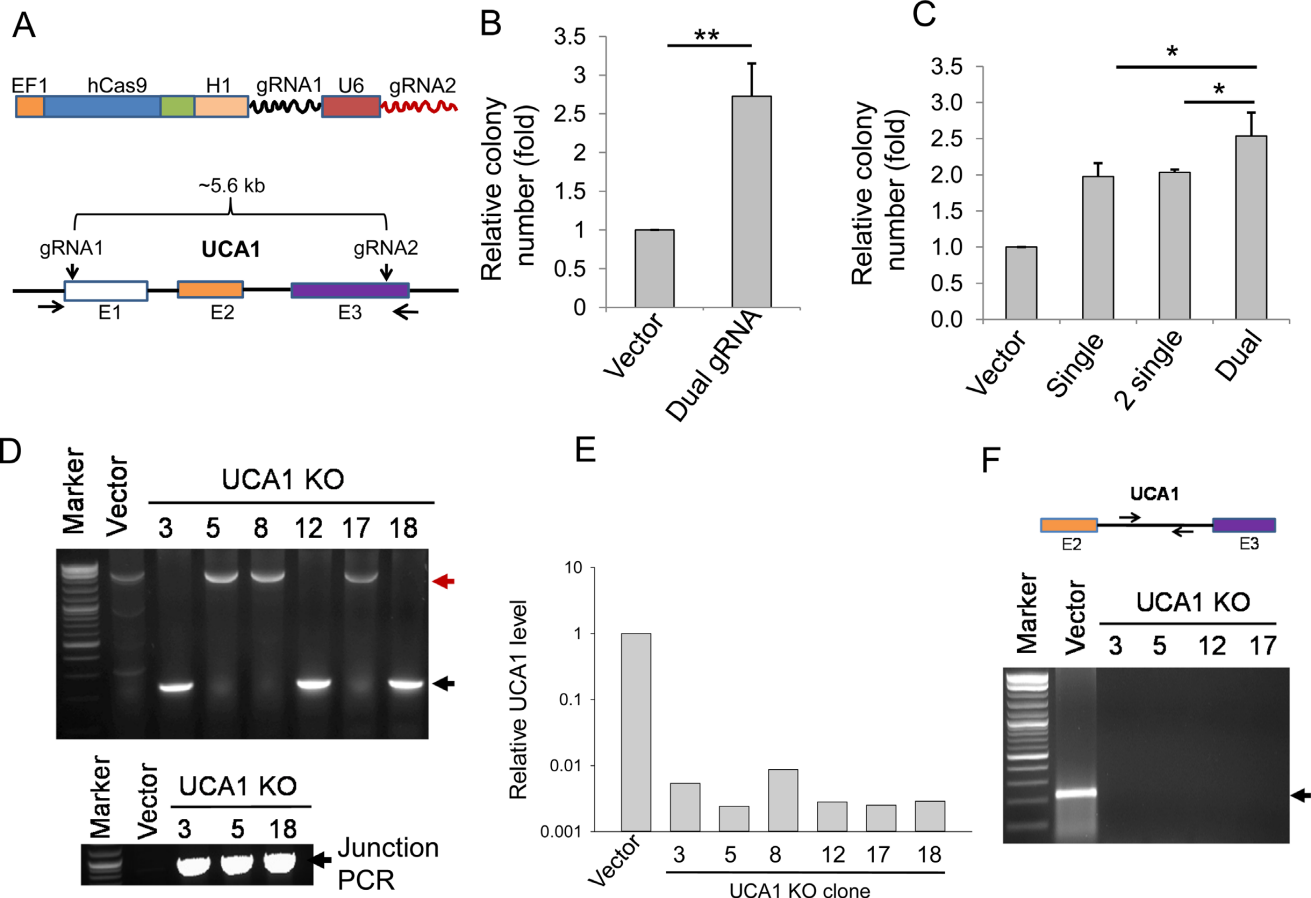
Targeting UCA1 with a dual gRNA approach. (**A**) Strategy for targeting UCA1 with dual gRNAs. (**B**) Colony formation for vector and UCA1 KO after puromycin selection. (**C**) The dual gRNA approach increases colony number, as compared to single or two individual gRNAs in combination. (**D**) Detection of UCA1 deletion by genomic PCR using primers UCA1-outside-5.5 and UCA1-outside-3.5. The red arrow (2.8 kb) indicates a PCR product corresponding to a replacement and the black arrow indicates a deletion (227 bp). Clones #3, #12 and #18 are expected to have a 2.8-kb fragment, but missing here possibly because a deletion band is much smaller, and thus it is more favorably amplified. Indeed, junction PCR with primers UCA1-Recom-5.1 and EF1–seq-3.2 revealed that clones #3, #5 and #12 all carried the HR fragment (bottom). (**E**) Detection of UCA1 expression in UCA1 KO clones by qRT-PCR. *P* value <0.01 for all six clones, as compared to vector control. (**F**) No PCR product (bottom) except for vector control (220 bp) was detected in UCA1 KO clones using primers derived from the region as indicated on top (UCA1-intron-5.1 and UCA1-intron-3.1). Values in (B) and (C) are means of ± SE (*n* = 3). *P < 0.05 and **P < 0.01.

To overcome this problem, we constructed a dual gRNA system (Figure 5A, top), which carries two tandem gRNAs, under H1 and U6 promoters, respectively, in the same single vector, allowing co-expression of two gRNAs and hCas9. While gRNA1 targeted toward 5′ end of UCA1 exon 1 (same as single gRNA as shown in Supplementary Figure S5A), gRNA2 targeted toward 3′ end of exon 3 such that almost the entire UCA1 sequences can be deleted through NHEJ or replaced by HR mechanism (∼5.6 kb; Figure 5A, bottom). In addition, we made a second single gRNA (same as gRNA2). Co-transfection of donor and UCA1 dual vector resulted in a 2.73-fold increase in colony number compared to control (Figure 5B). Of interest, the colony number from the UCA1 dual gRNA system was higher than either one single or two single gRNAs in combination (Figure 5C). To confirm knockout of UCA1, we first amplified the dual-gRNA-targeted region by genomic PCR using primers outside of two gRNAs (Figure 5A). Three (#3, #12 and #18) out of 20 randomly picked single colonies showed deletion bands, whereas there was no deletion band in other three clones (#5, #8 and #17) (Figure 5D). We then selected clones #3, #5 and #18 for junction PCR which revealed that they carried HR fragments (Figure 5D bottom). DNA sequencing indicated that they had identical sequences (Supplementary Figure S5C). In consistent with the genomic PCR results, although some background was amplified by sensitive qRT-PCR, we demonstrated a substantial reduction of UCA1 (Figure 5E). Finally, we picked four of these clones (3, 5, 12 and 17) representing a deletion and replacement, respectively, for genomic PCR using primers derived from the inside of targeted region (Figure 5F, top). It is evident that no product was detected for these four clones except for vector control (Figure 5F, bottom), suggesting that they are all complete knockouts.

We also tested two additional lncRNAs, lncRNA-21A and AK023948. We designed a dual gRNA targeting a 475-bp fragment covering the lncRNA-21A sequence (Supplementary Figure S6A). Co-transfection of donor and lncRNA-21A dual vectors into HCT-116 cells resulted in a 16.3-fold increase in colony number compared to control (Supplementary Figure S6B). Using primers derived from either outside or inside the lncRNA-21A dual-gRNA-targeted sides (Supplementary Figure S6C) we detected one copy was knockout in clone S7; genomic PCR showed that wild-type lncRNA-21A was completely lost in clones M2 and S38 (Supplementary Figure S6D). Junction PCR confirmed that all three clones carried HR fragments and they all had identical sequences, as determined by DNA sequencing (Supplementary Figure S6E). Similarly, we were able to knockout AK023948 in MCF-7 cells (Supplementary Figure S7). Therefore, our dual gRNA approach increases the targeting efficiency by CRISPR/Cas9 system, demonstrating its feasibility for targeting lncRNAs with either a short fragment (475 bp for lncRNA-21A) or a relatively long fragment (>5.6 kb for UCA1).

**Figure 6.**
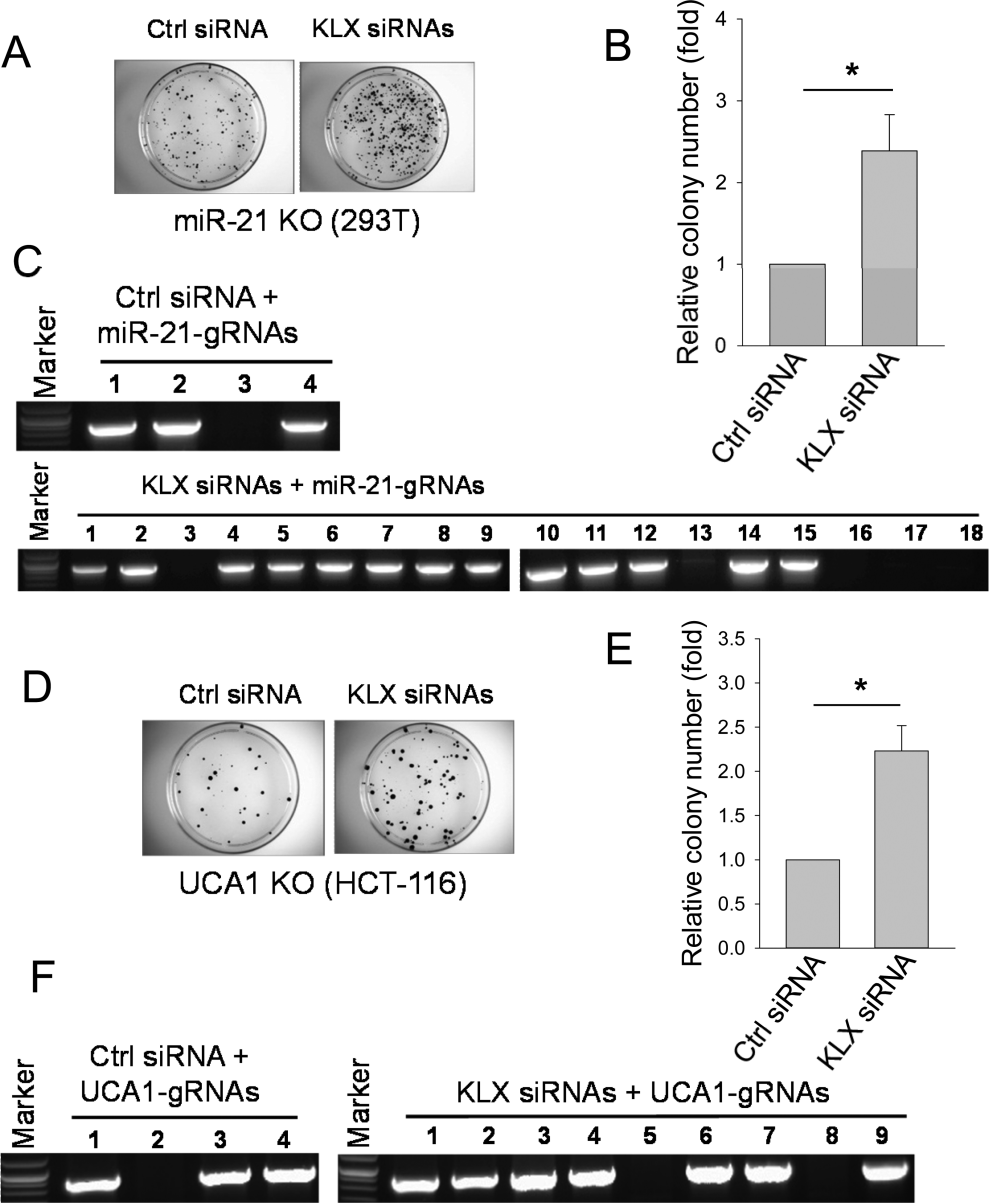
Enhancing the CRISPR/Cas9-mediated targeting efficiency by siRNAs against Ku70, Lig4 and XRCC4 (KLX siRNAs). (**A**) miR-21 KO in HEK293T cells with representative images of colonies. (**B**) Relative colony number for control siRNA or KLX siRNAs for miR-21 gRNAs. (**C**) Junction PCR products using primers miR-21-recom-5.2 and EF1-Seq-3.2. (**D**) UCA1 KO in HCT-116 cells with representative images of colonies. (**E**) Relative colony number for control siRNA or KLX siRNAs for UCA1-gRNAs. (**F**) Junction PCR products using primers UCA1-Recom-5.1 and EF1–seq-3.2. Pictures were taken 7 days after puromycin selection for both miR-21 and UCA1 KO. Values in (B) and (E) are means of ± SE (*n* = 3). *P < 0.05.

These findings further support the notion that established human cell lines have a strong NHEJ activity relative to HR because even under selection pressure, we are still able to see a significant frequency of deletion. Therefore, we asked if we can increase the HR targeting frequency by suppressing genes involved in NHEJ. We chose three key genes, Ku70, Lig4 and XRCC4, that have been implicated in NHEJ pathway (35–37). Thus, we used siRNA to transiently knockdown Ku70, Lig4 and XRCC4 simultaneously. First, we confirmed that the expressions of Ku70, Lig4 and XRCC4 were knockdown. The siRNAs resulted in at least 23.2% or 37.3% decrease in the expression of Ku70, Lig4 and XRCC4 compared to control siRNA in HEK293T cells (Supplementary Figure S8A–C) or HCT-116 cells (Supplementary Figure S8D–F). To determine effect of suppression of NHEJ pathway on the colony formation, we conducted either microRNA (miR-21 gRNAs) or lncRNAs (UCA1 dual gRNA and lncRNA-21A dual gRNA) transfection 24 h following the siRNA treatment. As anticipated, knockdown of Ku70, Lig4 and XRCC4 by siRNAs led to a 2.4-fold increase in Cas9-mediated colony number compared to control siRNA for miR-21 (Figure 6A and B). To determine whether the colony number reflects HR events, we performed junction PCR using primers miR-21-recom-5.2 derived from sequence outside the HR event and EF1-Seq-3.2 derived from the donor vector (Supplementary Figure S3A). DNA sequencing of the junction PCR products confirmed the HR events (Figure 6C). We obtained 75% HR rate for control siRNA and 78% HR rate for KLX siRNAs. Similarly, UCA1 dual gRNA knockout under the suppression of NHEJ pathway resulted in a 2.2-fold increase in colony number in HCT-116 cells (Figure 6D and E). DNA sequencing of the junction PCR products also indicated roughly the same HR rate for siRNA or KLX siRNAs (Figure 6F), suggesting that relative colony number can represent HR events. LncRNA-21A dual gRNA knockout following the transient knockdown of Ku70, Lig4 and XRCC4 resulted in a 1.2-fold increase in colony number in HCT-116 cells (Supplementary Figure S9). Together, these results suggest that the Cas9-medated targeting frequency through HR can be improved by suppressing the NHEJ pathway.

## DISCUSSION

Non-coding genes represent the vast majority of human transcripts. However, little is known about the physiological functions for most of them, especially lncRNAs. Thus, the present study aims to explore the applicability of CRISPR/Cas9 to non-coding genes for their functional studies. Unlike protein-coding genes, non-coding genes such as lncRNAs lack open reading frames, although their molecular weight is comparable to that of protein-coding genes. Because of this, the commonly used approaches of CRISPR/Cas9 system may have limited utility for knockout of non-coding genes. Here we demonstrate that a selection approach with a donor vector for HR can significantly increase targeting efficiency. In particular, the dual gRNA method allows us to target various sizes of a given DNA segment. Moreover, suppression of NHEJ pathway can further increase HR to improve target efficiency with our dual gRNA and donor vector. In addition to non-coding genes, this platform can also be applied to protein-coding genes. Given that about 30% of chance for any small insertion or deletion does not change the downstream open reading frames, screening effort for knockout clones can be significantly reduced by this approach. Finally, our platform can be easily adapted to other types of cells of different organisms.

Compared to lncRNAs, microRNAs are relatively easy to knock out. Our results suggest that the knockout efficiency of microRNAs is higher than that of lncRNAs (Figures 2A and 4B). This may be attributed to the fact that microRNA genes are much smaller in size than lncRNAs; mature microRNAs are ∼22 nucleotides in length. Of interest, although we target pre-miR-21, DNA sequencing reveals that alteration of the precursor can also lead to knockout of miR-21, which may be due to disruption of microRNA processing. On the other hand, lncRNAs are much larger than microRNAs. A previous study has shown that MALAT1 can be knocked out using ZFN or TALEN by introducing a poly A to terminate the downstream transcription (38). Although our donor vector also carries a poly A signal, apparently, this is not sufficient because a strong NHEJ activity can produce deletions or insertions. This ‘heterozygous’ feature is more prominent in polyploid cells, including most cancer cell lines.

Our results further suggest that HR combined with a donor vector can greatly increase the knockout efficiency. Due to the selection that kills non-transfected cells, transfection efficiency is no longer an important factor. This feature is critical because there are many cell lines with a relative low transfection even with different transfection reagents. Moreover, this HR system may reduce off-target frequency by CRISPR/Cas9 (39,40) because the donor is less likely to combine into the genome at off-target sites due to lack of homologous flanking DNA regions and thus, those cells carrying off-target sites, but without donor vector, are expected to be lost during puromycin selection.

Although we used FACS-sorted single cell colonies for most of our experiments, we noticed that manually picked single colonies in a 10 cm dish can also generate relatively pure clones as long as the starting cell density is low. The benefit of this approach is evident, i.e., cost saving and less stress to the cells.

One feature of this study is a dual gRNA approach which is especially useful for lncRNA knockout. For instance, we detected a significant portion of deletion with the dual gRNAs at the pre-designed sites. However, with a single gRNA or combination of two gRNAs in separate vectors, we rarely detected measurable deletions, as determined by genomic PCR. In consistent with this, targeting efficiency as measured by surviving colony number with the dual gRNA approach is higher than that with two single gRNAs which have the same target sequences. Although there are several possibilities for this, one of them may be that two gRNAs in the dual system are always present in the same cells. Additional advantage of using the dual gRNA system is the significant improvement of screening procedure. With appropriate primers, it is much easy and fast to identify potential knockout clones. For example, we are able to obtain at least 74% HR rate for miR-21, UCA1 and lncRNA-21A (Supplementary Figure S10).

Since our donor vector carries two loxP sites, it is possible to make a double or triple knockout sequentially. This can be accomplished by excising out the marker genes in the previous knockout such as miR-21 knockout (Figure 2B). Once excision, the cells are no longer green and they are sensitive to puromycin, which makes the selection and cell sorting possible again.

Finally, our results suggest that the targeting efficiency can be further improved by suppression of NHEJ pathways. This approach has been successfully studied in human somatic cells. For instance, suppression of Ku70 and XRCC4 by RNAi can greatly enhance gene targeting efficiency (41). We suppress three genes (Ku70, Lig4 and XRCC4) simultaneously and the targeting efficiency is significantly increased, suggesting that decreased efficiency in NHEJ-mediated knockout can potentially benefit the HR-mediated knockout. Nevertheless, it remains to be determined whether targeting efficiency can be further increased by knockdown of various NHEJ genes individually or in different combinations.

## SUPPLEMENTARY DATA

Supplementary Data are available at NAR Online.

SUPPLEMENTARY DATA

## References

[1] Ezkurdia I., Juan D., Rodriguez J.M., Frankish A., Diekhans M., Harrow J., Vazquez J., Valencia A., Tress M.L. (2014). Multiple evidence strands suggest that there may be as few as 19 000 human protein-coding genes. Hum. Mol. Genet.

[2] Xie C., Yuan J., Li H., Li M., Zhao G., Bu D., Zhu W., Wu W., Chen R., Zhao Y. (2014). NONCODEv4: exploring the world of long non-coding RNA genes. Nucleic Acids Res..

[3] Zamore P.D., Tuschl T., Sharp P.A., Bartel D.P. (2000). RNAi: double-stranded RNA directs the ATP-dependent cleavage of mRNA at 21 to 23 nucleotide intervals. Cell.

[4] Fatica A., Bozzoni I. (2014). Long non-coding RNAs: new players in cell differentiation and development. Nat. Rev. Genet..

[5] Le Provost F., Lillico S., Passet B., Young R., Whitelaw B., Vilotte J.L. (2010). Zinc finger nuclease technology heralds a new era in mammalian transgenesis. Trends Biotechnol..

[6] van der Oost J. (2013). Molecular biology. New tool for genome surgery. Science.

[7] Kim Y.G., Cha J., Chandrasegaran S. (1996). Hybrid restriction enzymes: zinc finger fusions to Fok I cleavage domain. Proc. Natl Acad. Sci. U.S.A..

[8] Wright D.A., Li T., Yang B., Spalding M.H. (2014). TALEN-mediated genome editing: prospects and perspectives. Biochem. J..

[9] Ding Q., Regan S.N., Xia Y., Oostrom L.A., Cowan C.A., Musunuru K. (2013). Enhanced efficiency of human pluripotent stem cell genome editing through replacing TALENs with CRISPRs. Cell Stem Cell.

[10] Hwang W.Y., Fu Y., Reyon D., Maeder M.L., Tsai S.Q., Sander J.D., Peterson R.T., Yeh J.R., Joung J.K. (2013). Efficient genome editing in zebrafish using a CRISPR-Cas system. Nat. Biotechnol..

[11] Chang N., Sun C., Gao L., Zhu D., Xu X., Zhu X., Xiong J.W., Xi J.J. (2013). Genome editing with RNA-guided Cas9 nuclease in zebrafish embryos. Cell Res..

[12] Wang H., Yang H., Shivalila C.S., Dawlaty M.M., Cheng A.W., Zhang F., Jaenisch R. (2013). One-step generation of mice carrying mutations in multiple genes by CRISPR/Cas-mediated genome engineering. Cell.

[13] Shen B., Zhang J., Wu H., Wang J., Ma K., Li Z., Zhang X., Zhang P., Huang X. (2013). Generation of gene-modified mice via Cas9/RNA-mediated gene targeting. Cell Res..

[14] Bhaya D., Davison M., Barrangou R. (2011). CRISPR-Cas systems in bacteria and archaea: versatile small RNAs for adaptive defense and regulation. Annu. Rev. Genet..

[15] Barrangou R., Horvath P. (2012). CRISPR: new horizons in phage resistance and strain identification. Annu. Rev. Food Sci. Technol..

[16] Jinek M., Chylinski K., Fonfara I., Hauer M., Doudna J.A., Charpentier E. (2012). A programmable dual-RNA-guided DNA endonuclease in adaptive bacterial immunity. Science.

[17] Dicarlo J.E., Norville J.E., Mali P., Rios X., Aach J., Church G.M. (2013). Genome engineering in Saccharomyces cerevisiae using CRISPR-Cas systems. Nucleic Acids Res..

[18] Cho S.W., Kim S., Kim J.M., Kim J.S. (2013). Targeted genome engineering in human cells with the Cas9 RNA-guided endonuclease. Nat. Biotechnol..

[19] Cong L., Ran F.A., Cox D., Lin S., Barretto R., Habib N., Hsu P.D., Wu X., Jiang W., Marraffini L.A. (2013). Multiplex genome engineering using CRISPR/Cas systems. Science.

[20] Jiang W., Bikard D., Cox D., Zhang F., Marraffini L.A. (2013). RNA-guided editing of bacterial genomes using CRISPR-Cas systems. Nat. Biotechnol..

[21] Mali P., Yang L., Esvelt K.M., Aach J., Guell M., DiCarlo J.E., Norville J.E., Church G.M. (2013). RNA-guided human genome engineering via Cas9. Science.

[22] Gratz S.J., Cummings A.M., Nguyen J.N., Hamm D.C., Donohue L.K., Harrison M.M., Wildonger J., O'Connor-Giles K.M. (2013). Genome engineering of Drosophila with the CRISPR RNA-guided Cas9 nuclease. Genetics.

[23] Xiao A., Wang Z., Hu Y., Wu Y., Luo Z., Yang Z., Zu Y., Li W., Huang P., Tong X. (2013). Chromosomal deletions and inversions mediated by TALENs and CRISPR/Cas in zebrafish. Nucleic Acids Res..

[24] Han J., Zhang J., Chen L., Shen B., Zhou J., Hu B., Du Y., Tate P.H., Huang X., Zhang W. (2014). Efficient in vivo deletion of a large imprinted lncRNA by CRISPR/Cas9. RNA Biol..

[25] Sachdeva M., Zhu S., Wu F., Wu H., Walia V., Kumar S., Elble R., Watabe K., Mo Y.Y. (2009). p53 represses c-Myc through induction of the tumor suppressor miR-145. Proc. Natl Acad. Sci. U.S.A..

[26] Ayene I.S., Ford L.P., Koch C.J. (2005). Ku protein targeting by Ku70 small interfering RNA enhances human cancer cell response to topoisomerase II inhibitor and gamma radiation. Mol. Cancer Ther..

[27] Muylaert I., Elias P. (2007). Knockdown of DNA ligase IV/XRCC4 by RNA interference inhibits herpes simplex virus type I DNA replication. J. Biol. Chem..

[28] Si M.L., Zhu S., Wu H., Lu Z., Wu F., Mo Y.Y. (2007). miR-21-mediated tumor growth. Oncogene.

[29] Han Y.C., Park C.Y., Bhagat G., Zhang J., Wang Y., Fan J.B., Liu M., Zou Y., Weissman I.L., Gu H. (2010). microRNA-29a induces aberrant self-renewal capacity in hematopoietic progenitors, biased myeloid development, and acute myeloid leukemia. J. Exp. Med..

[30] Huang J., Zhou N., Watabe K., Lu Z., Wu F., Xu M., Mo Y.Y. (2014). Long non-coding RNA UCA1 promotes breast tumor growth by suppression of p27 (Kip1). Cell Death Dis..

[31] Pagano A., Castelnuovo M., Tortelli F., Ferrari R., Dieci G., Cancedda R. (2007). New small nuclear RNA gene-like transcriptional units as sources of regulatory transcripts. PLoS Genet..

[32] He H., Nagy R., Liyanarachchi S., Jiao H., Li W., Suster S., Kere J., de la Chapelle A. (2009). A susceptibility locus for papillary thyroid carcinoma on chromosome 8q24. Cancer Res..

[33] Zhang Z., Zhu Z., Watabe K., Zhang X., Bai C., Xu M., Wu F., Mo Y.Y. (2013). Negative regulation of lncRNA GAS5 by miR-21. Cell Death Differ..

[34] Asangani I.A., Rasheed S.A., Nikolova D.A., Leupold J.H., Colburn N.H., Post S., Allgayer H. (2008). MicroRNA-21 (miR-21) post-transcriptionally downregulates tumor suppressor Pdcd4 and stimulates invasion, intravasation and metastasis in colorectal cancer. Oncogene.

[35] Grundy G.J., Moulding H.A., Caldecott K.W., Rulten S.L. (2014). One ring to bring them all—the role of Ku in mammalian non-homologous end joining. DNA Repair.

[36] Felgentreff K., Du L., Weinacht K.G., Dobbs K., Bartish M., Giliani S., Schlaeger T., DeVine A., Schambach A., Woodbine L.J. (2014). Differential role of nonhomologous end joining factors in the generation, DNA damage response, and myeloid differentiation of human induced pluripotent stem cells. Proc. Natl Acad. Sci. U.S.A..

[37] Francis D.B., Kozlov M., Chavez J., Chu J., Malu S., Hanna M., Cortes P. (2014). DNA Ligase IV regulates XRCC4 nuclear localization. DNA Repair.

[38] Gutschner T., Hammerle M., Eissmann M., Hsu J., Kim Y., Hung G., Revenko A., Arun G., Stentrup M., Gross M. (2013). The noncoding RNA MALAT1 is a critical regulator of the metastasis phenotype of lung cancer cells. Cancer Res..

[39] Lin Y., Cradick T.J., Brown M.T., Deshmukh H., Ranjan P., Sarode N., Wile B.M., Vertino P.M., Stewart F.J., Bao G. (2014). CRISPR/Cas9 systems have off-target activity with insertions or deletions between target DNA and guide RNA sequences. Nucleic Acids Res..

[40] Shen B., Zhang W., Zhang J., Zhou J., Wang J., Chen L., Wang L., Hodgkins A., Iyer V., Huang X. (2014). Efficient genome modification by CRISPR-Cas9 nickase with minimal off-target effects. Nat. Methods.

[41] Bertolini L.R., Bertolini M., Maga E.A., Madden K.R., Murray J.D. (2009). Increased gene targeting in Ku70 and Xrcc4 transiently deficient human somatic cells. Mol. Biotechnol..

