# Supplementary material for: Targeting non-coding RNAs with the CRISPR/Cas9 system in human cell lines
Source: Nucleic Acids Res. 2014 Nov 20;43(3):e17. doi: 10.1093/nar/gku1198 (PMC4330338; doi:10.1093/nar/gku1198)
Supplement: SUPPLEMENTARY DATA [file supp_43_3_e17__index.html]

Targeting non-coding RNAs with the CRISPR/Cas9 system in human cell lines — Targeting non-coding RNAs with the CRISPR/Cas9 system in human cell lines — SUPPLEMENTARY DATA 

# Targeting non-coding RNAs with the CRISPR/Cas9 system in human cell lines

## SUPPLEMENTARY DATA

**Files in this Data Supplement:**

- SUPPLEMENTARY DATA
