# Supplementary material for: Targeting non-coding RNAs with the CRISPR/Cas9 system in human cell lines
Source: Nucleic Acids Res. 2014 Nov 20;43(3):e17. doi: 10.1093/nar/gku1198 (PMC4330338; doi:10.1093/nar/gku1198)
Supplement: SUPPLEMENTARY DATA [file supp_gku1198_nar-02186-met-h-2014-File003.pdf]

Supplementary Materials

Supplementary Table 1, Primers used in this study

miR-21-T1(gRNA1)	ATGTTGACTGTTGAATCTCA
miR-21-T2(gRNA2)	CTCATGGCAACACCAGTCGA
miR-21-T3(gRNA3)	ATGTCAGACAGCCCATCGAC
miR-21-5.1	GGTTCGATCTTAACAGGCCAG
miR-21-3.1	GAGATGAACCACGACTAGAGG
miR-21-L-Spe-5.1	GGCCAGTGAAACTAGTGTCTTACAAGTGAGCTGACACC
miR-21-L-Bsa1-3.1	GCTATACGAAGTTATAGGTGGTACAGCCATGGAGAT
miR-21-R-Bbs-5.2	CATTATACGAAGTTATTTTGGTATCTTTCATCTGACCATCC
miR-21-R-Sal-3.2	TATCACCGGTGTCGACCAAGAAAGGAAGGCACGAACAG
miR-21-recom-5.2	ACCCTCTGGGGAAATTGATC

UCA1-T1(gRNA1)	GTGCATGGTGGAGAGATGAT
UCA1-E3-T1(gRNA2)	TTCTGGAATGGTGAACCCAA
UCA1-inside-5.3	CCTTAACTAATTAACCCACC
UCA1-inside-3.3	AAGAGAGTCAGCGAAGGGAG
UCA1-intron1-5.1	CTTTTCTCCAGCCTCTGTCG
UCA1-intron1-3.1	CAGACACCAGTGGAATGTGG
UCA1-RT-5.2	CTCCCTCCTCTGCTGACAAC
UCA1-RT-3.2	GCACCAAGTGTCAAGCATGT
UCA1-outside-5.5	AGTGAAACAGCCAATTCCTG
UCA1-outside-3.5	TGATCGGCTCTCGGCCTAAT
UCA1-left-BamH1-5.1	GCTCTAGAACTAGTGGATCCGTCAGATCAGGTCATCCTAT
UCA1-left-BamH1-3.1	GCTATACGAAGTAGGGATCCAAGAATGTCACAGGGTCGAT
UCA1-right-R1-5.2	TTATACGAAGTTATGAATTCTAAAGCCATGCCCATCAGAC
UCA1-right-R1-3.2	ATAAGCTTGATATCGAATTCTTGAGCTTGGAACTGCCCTA
UCA1-recom-5.1	CCGGCAAGTCTGACTCAAGT

21A-dual-T1	TATTTCTTCCATCACCATAA
21A-dual-T2	AAGTGTTTGAATTAGCAGGT
21A-left-BamH1-5.1	GCTCTAGAACTAGTGGATCCtgggtagtctgagtccagaa
21A-left-BamH1-3.1	GCTATACGAAGTAGGGATCCcacacttggtcaactattta
21A-right-R1-5.1	TTATACGAAGTTATGAATTCgtagtgagctttgattgcat
21A-right-R1-3.1	ATAAGCTTGATATCGAATTCaggttaaatgagatggcatg
21A-outside-5.1	GAATGAATGAATGAACAGCT
21A-outside-3.1	AGATGACCTAAGAAATTTGG
21A-recom-5.1A	CCATCTGCGTCTCCTTTTCG

AKO-dual-T1-5.1	GAGTTTTAGTCACCTATCTA
AK0-dual-I-T1-3.1	GGTGATCCTTGTGCACGGCC
AK023948-RT-5.1	CCCACAAAGCTCTTTTCTGC
AK023948-RT-3.1	GGTGCCCAAGTAAAGCACAT
AK0-outside-5.1	TTTTAAGCAAGGCCTGGTGC
AK0-outside-3.1	TCAGCTCAAGCTCCTATGGC
AK0-outside-5.2	TCAGTCACTCCCACTTATAA
AK0-outside-3.2	TTAGGCCAATGTGCAATTAG
AK0-right-I-R1-5.1	TTATACGAAGTTATGAATTCGGTGCCTCTCTGGCACTGAA
AK0-right-I-R1-3.1	ATAAGCTTGATATCGAATTCAGGTGGAACAGAGCATGTGC
AK023948-left-BamH1-5.1	GCTCTAGAACTAGTGGATCCTCTACTCCTAGAACTCCTTA

AK023948-left-BamH1-3.1	GCTATACGAAGTAGGGATCCCGACTAGTTTTATTAGACAG
AKO-inside-5.1	CAAATTACAGAACTACCAGT
AKO-inside-3.1	TGAGCTTCCAGTAATGTTCC
AK0-RT-5.1	CCCACAAAGCTCTTTTCTGC
AK0-RT-3.1	GGTGCCCAAGTAAAGCACAT
Gas5-RT-5.2	CAGTGTGGCTCTGGATAGCA
Gas5-RT-3.2	TTAAGCTGGTCCAGGCAAGT
EF1-Seq-3.2	ACCGGAGCGATCGCAGATCC

Fig. S1

Pre-miR-21 (WT)

TGTCGGGTAGCTTATCAGACTGATGTTGACTGTTGAATCTCATGGCAACACCAGTCGATGGGCTGTCTGACAT

KO#3

1:

TGTCGGGTAGCTTATCAGACTGATGTTGACTGTTGAATCTCATG
G~~caacaccagtcgatgg~~GCTGTCTGACAT

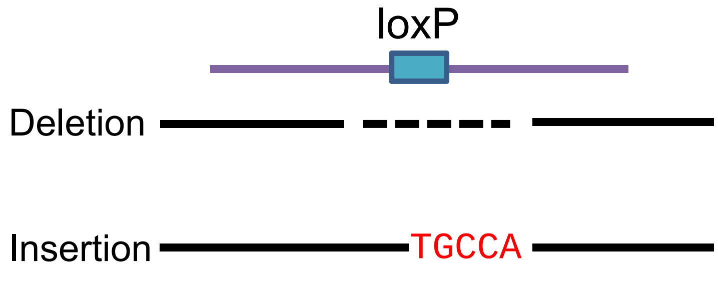

2:

TGTCGGGTAGCTTATCAGACTGATGTTGACTGTTGAATCTCATG
GCAACACCAGTTGCCAGATGGGCTGTCTGACAT

KO#4

1:

TGTCGGGTAGCTTATCAGACTGATGTTGACTGTCGAATCTC~~atg~~
~~gcaacaccagtcg~~ATGGGCTGTCTGACAT

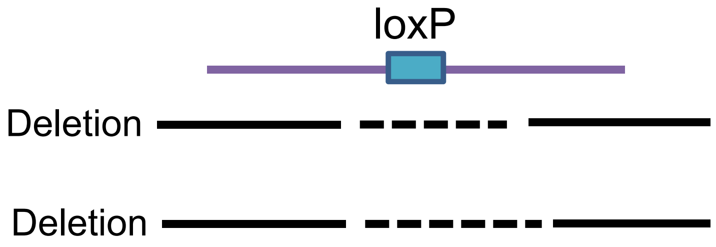

2:

TGTCGGGTAGCTTATCAGACTGATGTTGACTGTTGAATCTCATG
G~~caacaccagtcgatgggctgtctgacattttgg~~TATCTT

KO#5

TGTCGGGTAGCTTATCAGACTGATGTTGACTGTTGAATCTCATG
GCAACACCAGTCGGGCGCACGCGCTGGGTGTTCCCGCCTAGT
GACACTGGGCCCGCGATTCCTTGGAGCGGGTTGATGACGTCAGC
GTTCGAATTACCGATGGGCTGTCTGACAT

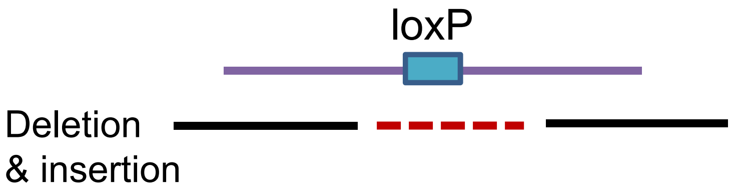

Fig. S2

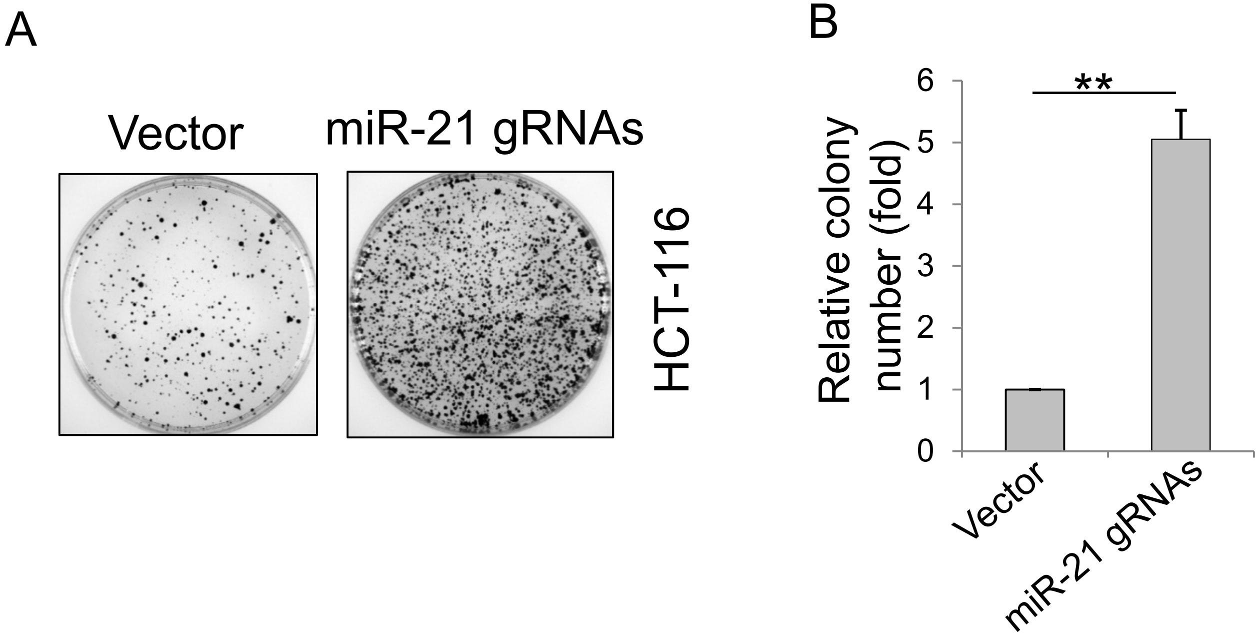
A
Vector
miR-21 gRNAs
HCT-116
B
**
Relative colony number (fold)
0
1
2
3
4
5
6
Vector
miR-21 gRNAs

Fig. S2

Pre-miR-21 (WT)

TGTCGGGTAGCTTATCAGACTGATGTTGACTGTTGAATCTCATGGCAACACCAGTCGATGGGCTGTCTGACAT

C

WT (238 bp)

TATGAGCATTATGTCAGAATAGAATAGAATTGGGGTTCGATCTTAACAGGCCAGAAATGCCTGGGTTTTTTTTGGTTTGTTTT
TGTTTTTGTTTTTTTATCAAATCCTGCCTGACTGTCTGCTTGTTTTGCCTACCATCGTGACATCTCCATGGCTGTACCACCTTGT
CGGGTAGCTTATCAGACTGATGTTGACTGTTGAATCTCATGGCAACACCAGTCGATGGGCTGTCTGACAT

KO #2 (235 bp)

TATGAGCATTATGTCAGAATAAGTCCCTATAGAGCTTTTTTTTGGCCCATTTGTTTATAAATATTTGTTTGCCTCTGGATAGTCT
CATCTTCTTCAAGTTGTGCCTACCTCCTCCAACATGATGCTCTGTTTGCCATTTGCACAAAACATCTTATGTTTGTCAATCGAG
ATGTATTTCCCACTCTCATTCATCCTGTCTTGCACTTAAGGACTATGTCGATGGGCTGTCTGACAT

KO #17 (202 bp)

TATGAGCATTATGTCAGAATAGAATAGAATTGGGGTTCGATCTTAACAGGCCAGAAATGCCTGGGTTTTTTTTGGTTTGTTTT
TGTTTTTGTTTTTTTATCAAATCCTGCCTGACTGTCTGCTTGTTTTGCCTACCATCGTGACATCTCCATGGCTGTACCACCTTGT
CGGGTAGCTTATCGT~~AACTGATGTTGACTGTTGAATCTCATGGCAACACCA~~CGATGGGCTGTCTGACAT

D

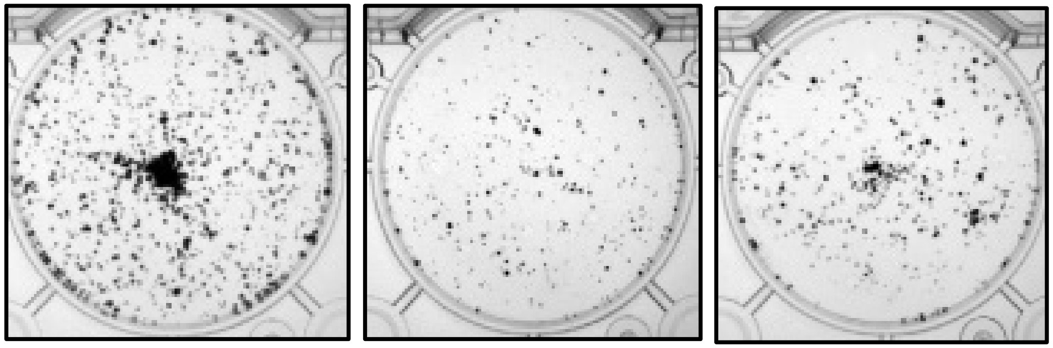

Vector | miR-21 KO #2 | miR-21 KO #17

HCT-116

Fig. S3

A

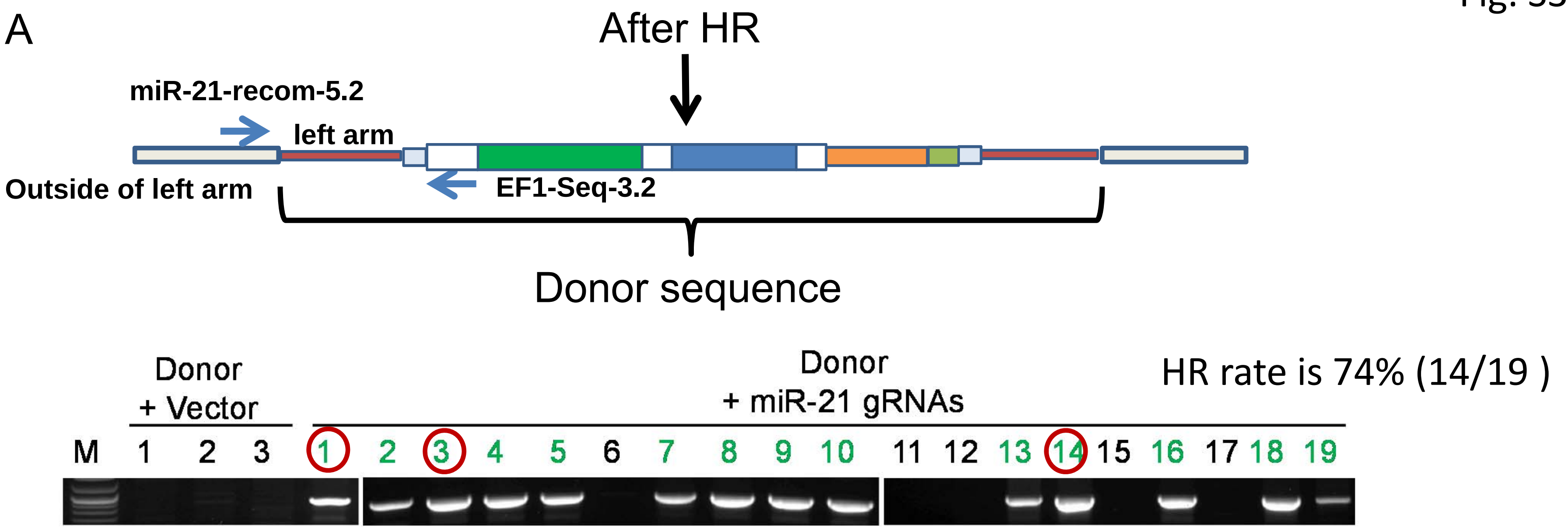

B

DNA sequences of junction PCR products for miR-21 knockout

Outside (miR-21-recom-5.2)

Left arm

ACCCTCTGGGGAAATTGATCTTTAAATTTTGAGACAGTATAAGGAAAATCTGGTTGGT
GTCTTACAAGTGAGCTGACACCATTTTTTATTCTGTGTATTTAGAATGAAGTCTTGAAAAAAACTTTATA
AAGACATCTTTAATCATTCCAAAATTGTGTCCGTTTTCTTGAGCGTTTTGATTTTTTACTTTTAGCTTAT
ACCAGCTGAATGGCAGCCTTGCCTAATCCACCTACAACAAGAATTTCTTAAGCTTTCTTTTATTTGCATG
AGAGAGCCACTACCAAGGCATGTTTTGTTATGCTGAAACTGGGCTGCTGCATACTGCTAAATGGCACCTC
TGGGATTGGCCTACCTGGGGATTTCTTGGTTTGTGAAAACAGGAGAGGAGAAATATCTCATACAAGTGAA
AGGATACTGGAGAGAGAAATTACCCATTTCTAAAAAAAAACCACACTCTGTCGTATCTGTGTTAATGTTT
TCTAGCATGTACTCTGGTTTCAACAGACACAAATTTATATGTTAACCCAGTTTTCTTGCCGTTCTGTAAG
TGTTTTATTCTTAGTGTGATTTTTTTCCATTGGGATGTTTTTGATTGAACTTGTTCATTTTGTTTTGCTT
GGGAGGAAAATAAACAATTTTACTTTTTTCCTTTAGGAGCATTATGAGCATTATGTCAGAATAGAATAGA
ATTGGGGTTCGATCTTAACAGGCCAGAAATGCCTGGGTTTTTTTGGTTTGTTTTTGTTTTTGTTTTTTTA
TCAAATCCTGCCTGACTGTCTGCTTGTTTTGCCTACCATCGTGACATCTCCATGGCTGTACCACCTATAA
CTTCGTATAGCATACATTATACGAAGTTATGCTAGC**AAGGATCTGCGATCGCTCCGGT**

Donor vector

EF1-Seq-3.2

Fig. S4

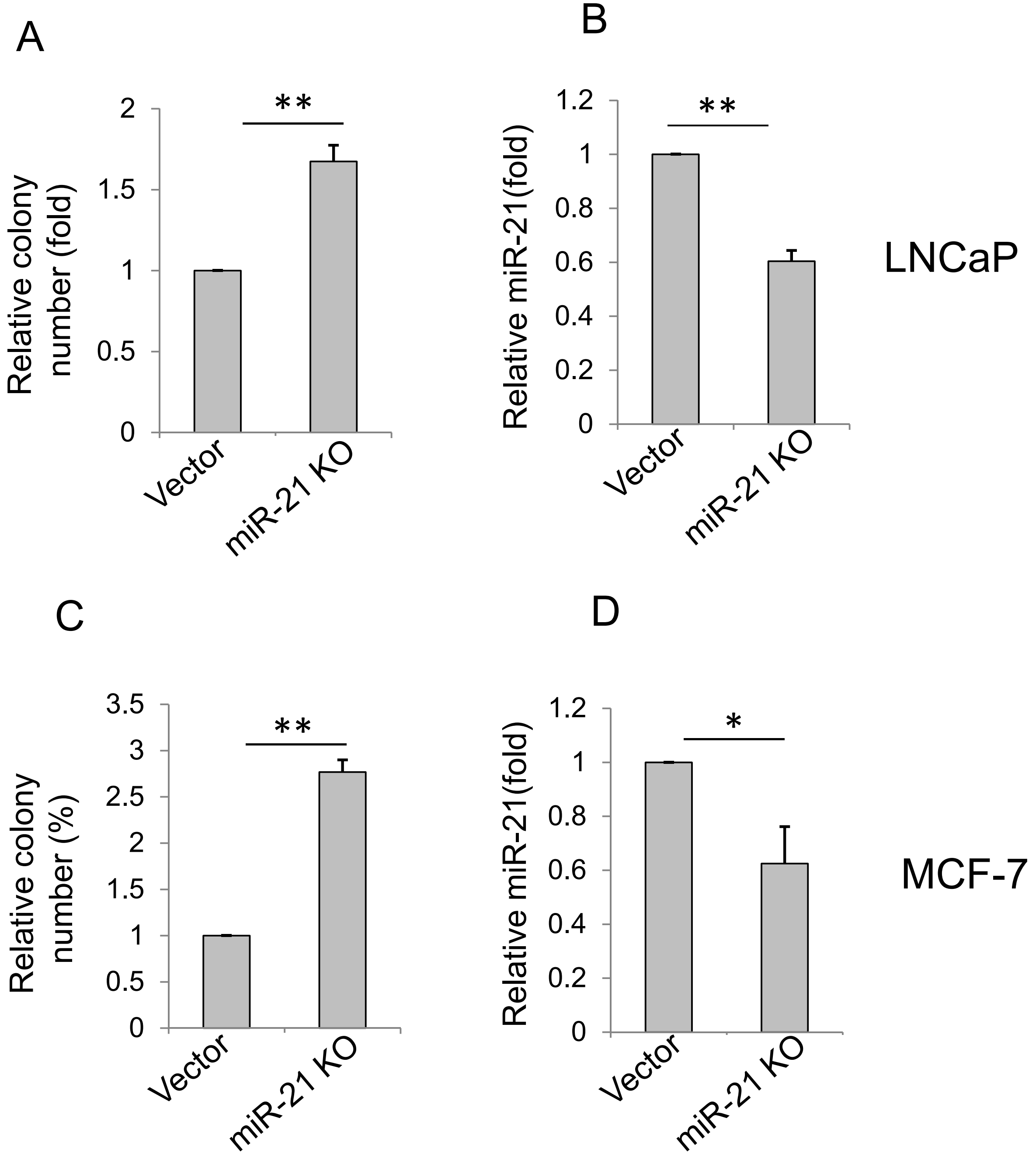

A
Relative colony number (fold)
2
1.5
1
0.5
0
**
Vector
miR-21 KO
B
Relative miR-21(fold)
1.2
1
0.8
0.6
0.4
0.2
0
**
Vector
miR-21 KO
LNCaP
C
Relative colony number (%)
3.5
3
2.5
2
1.5
1
0.5
0
**
Vector
miR-21 KO
D
Relative miR-21(fold)
1.2
1
0.8
0.6
0.4
0.2
0
*
Vector
miR-21 KO
MCF-7

Fig. S5

A

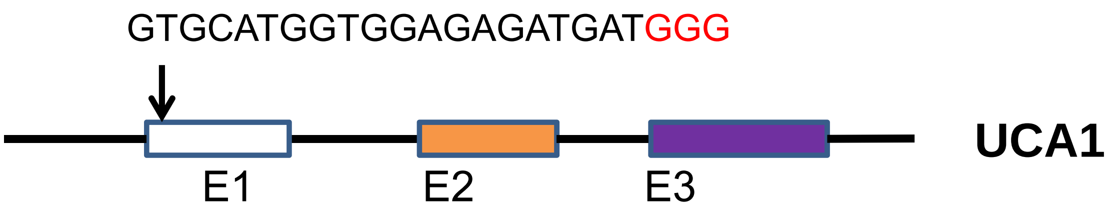

B

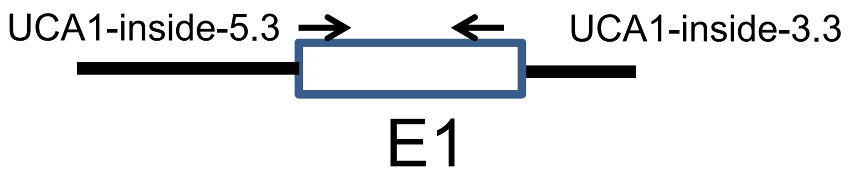

C

Outside (UCA1-recom-5.1)

Left arm

CCGGCAAGTCTGACTCAAGTGTCAGATCAGGTCATCCTATATAACCTCAGACATGCCCCAAACCCTGTGC
TGCTCACATATATACTCAGTGACCACCCTCCCAGAAGACACTGCATTGTGTGCGTTTCAGCTTTTTGCGT
CACCTCAGTGAAGGTGGTTTTCCTGTCCTTAGCCATAGTTTTCTCCCTTCACTAGGCTAAGAGGATTCAA
AGGACAGGGATCATTCCAGTCTTTTCCCTTCAATAGCAGCATCTGAGTGGCTGACCAGAGAGGTATTTCC
AAGAAGTGAAAACTGGGCTTGGGGTGAGAAAAGCTAAATGTCCTAAGGGGTTTCCTTTTAGATGACGGAG
GGAGATACCAGGGGACTCCTCCCTCCCGGGAGGTAGGGGGAGGGGGTGCACCCGGCGGCTCCCCTGAGAG
GCCCCACAGGCACATCTCAGGCTGTCCTCTGGGAAGAAATGACCCAGGAGCTGATATTCATGACCCTCCA
AGAGACCCGGCAGCTTGTGGGCATCTCAGATGAGCCCCAGGCAGTTCTGAAACCCAGGACCAGGAAAAGA
TAAGAGGGTGGGCTGAGGTCACATCACCCTGTAACGTTTCCAAAAGGGAACTGTCAGGCCTCTGAGCCGA
AGCTCAGCCATTGTAACCCCTGTGACCTGCACATATATGTCCAGGTGGCCTACAGGAGCCAAGAAGTCTG
GAGCAGCTGAAAAACAAGGAAGTGAAACAGCCAATTCCTGCCTTAACTAATTAACCCACCTTACGACATT
CCACCATTATGACGTGTTCCTGCCCTGCCCCAACTGATCAATCGACCCTGGGATCCCTACTTCGTATAGC
ATACATTATACGAAGTTAT**AAGGATCTGCGATCGCTCCGGT**

Donor vector

EF1-Seq-3.2

Fig. S6

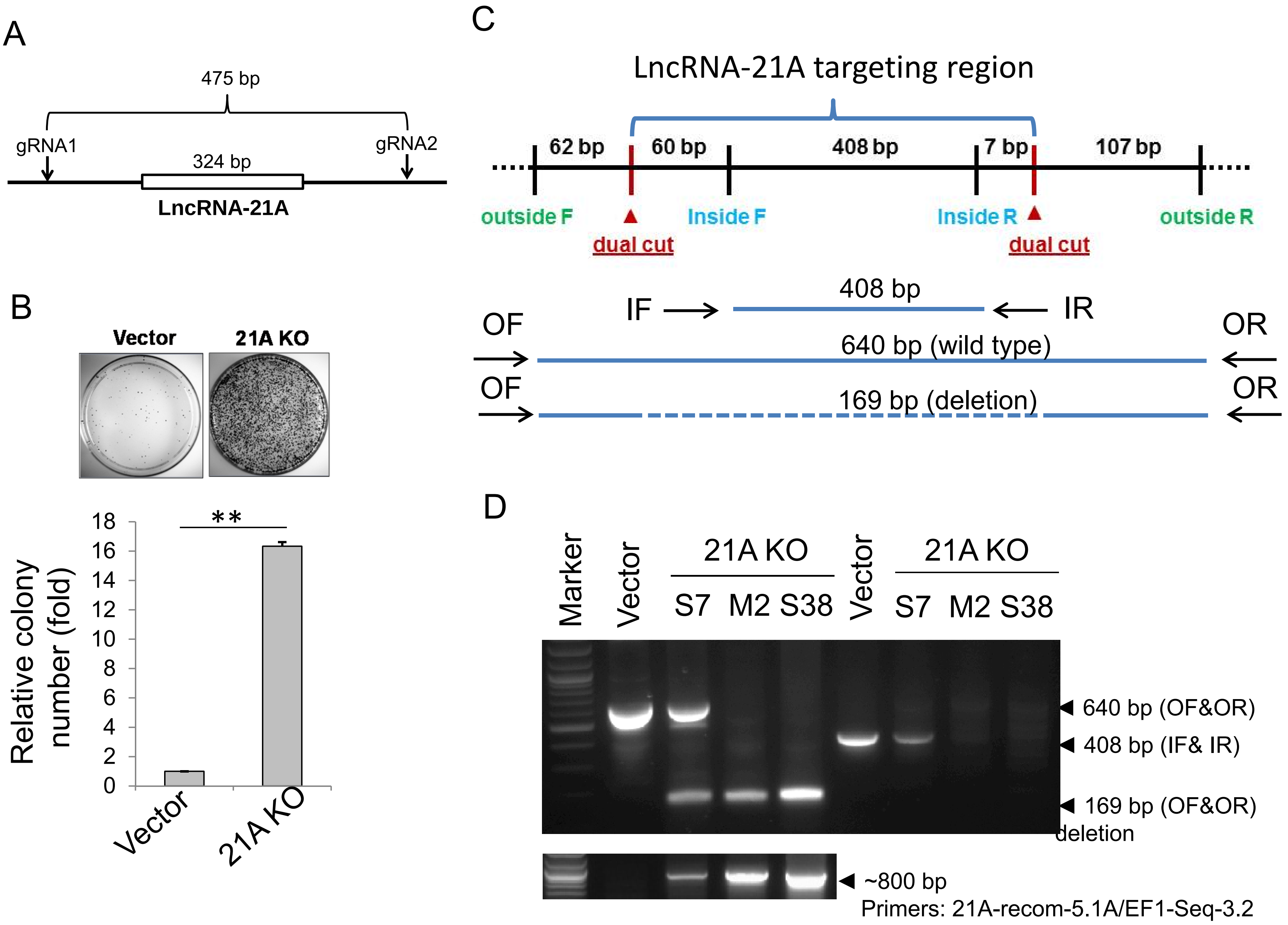

A
475 bp
gRNA1
gRNA2
324 bp
LncRNA-21A
B
Vector
21A KO
Relative colony number (fold)
18
16
14
12
10
8
6
4
2
0
**
Vector
21A KO
C
LncRNA-21A targeting region
62 bp
60 bp
408 bp
7 bp
107 bp
outside F
dual cut
Inside F
Inside R
dual cut
outside R
408 bp
IF
IR
OF
OR
640 bp (wild type)
OF
OR
169 bp (deletion)
D
Marker
Vector
21A KO
S7 M2 S38
Vector
21A KO
S7 M2 S38
640 bp (OF&OR)
408 bp (IF& IR)
169 bp (OF&OR) deletion
~800 bp
Primers: 21A-recom-5.1A/EF1-Seq-3.2

E

Outside (21A-recom-5.1A)

Left arm

CCATCTGCGTCTCCTTTTCGGTGTTGAAAGCTGGTAAGTGAGGACACCAGGATTGGAACCTGGGTAGTCT
GAGTCCAGAATCTCTATTTTCAAGTCTTCCTGCTCTCTGCTTCTGGCAAGTTTGATGTCCACTTTTGATC
TTCACCTACATTCCAGCATAATAGCTACTTTTGGTTGTTTTCTCAGCAGCACAAGAGAAGTGTGGCGAGA
TTTTTAGGTGAGTCATCTAGAGAAGTTAATCTTATTTTGGGAATTCTACTGGCAGCTTCAGGTGGGGAAA
ATTTTGTTATTTTCTATCCTCCTCTAGGTTCTAAAAGGGAAGAAAGATGGTGAGCGTAGAAAGATGTGAC
TGTATTCACTATTCACCCTTTGTCGGGTGGTGAGTAAGCAGCTTGCAAAAGCAATGAAGTTTGGAACAAT
CCAGAGAACCAAACTTTCAGCTGCCAGAGATGGCACCTGGTATCCTGGGTACATCTGCCTGTAGGGCCCA
GAAAGAGCTGGAAGCCAAGTGCATGGATCAGGTCTGTAGGAAGGTGGGAGAGCCAGGAATCGAGTGTCAG
GGGGCATTTATTACCCATGGAAGCAGGTTTTTGTCAATTTTGTTCACTGCTGGATCACTAACACCTGGAC
TGGTGCCTGGCCAGGTGGTGGCTTCATAATCATTTGTTGAGTGAATCAATGAATGAATGAATGAACAGCT
GTAGCAGATGCTAGCAGGGCTTCCTATTTCTTCCATCACCATAAAGGTGAAAGACATCATAAACGGGAAT
TTAGACAATCCTCAGAAATTTTCAACTGCCATGTATCTTGACTTGATGCTTCTAGTAGTTATATTTATTT
GTAATTCAATCTTTCTTTTTAAATAGTTGACCAAGTGTGGGATCCCTACTTCGTATAGCATACATTATAC
GAAGTTAT**AAGGATCTGCGATCGCTCCGGT**

Donor vector

EF1-Seq-3.2

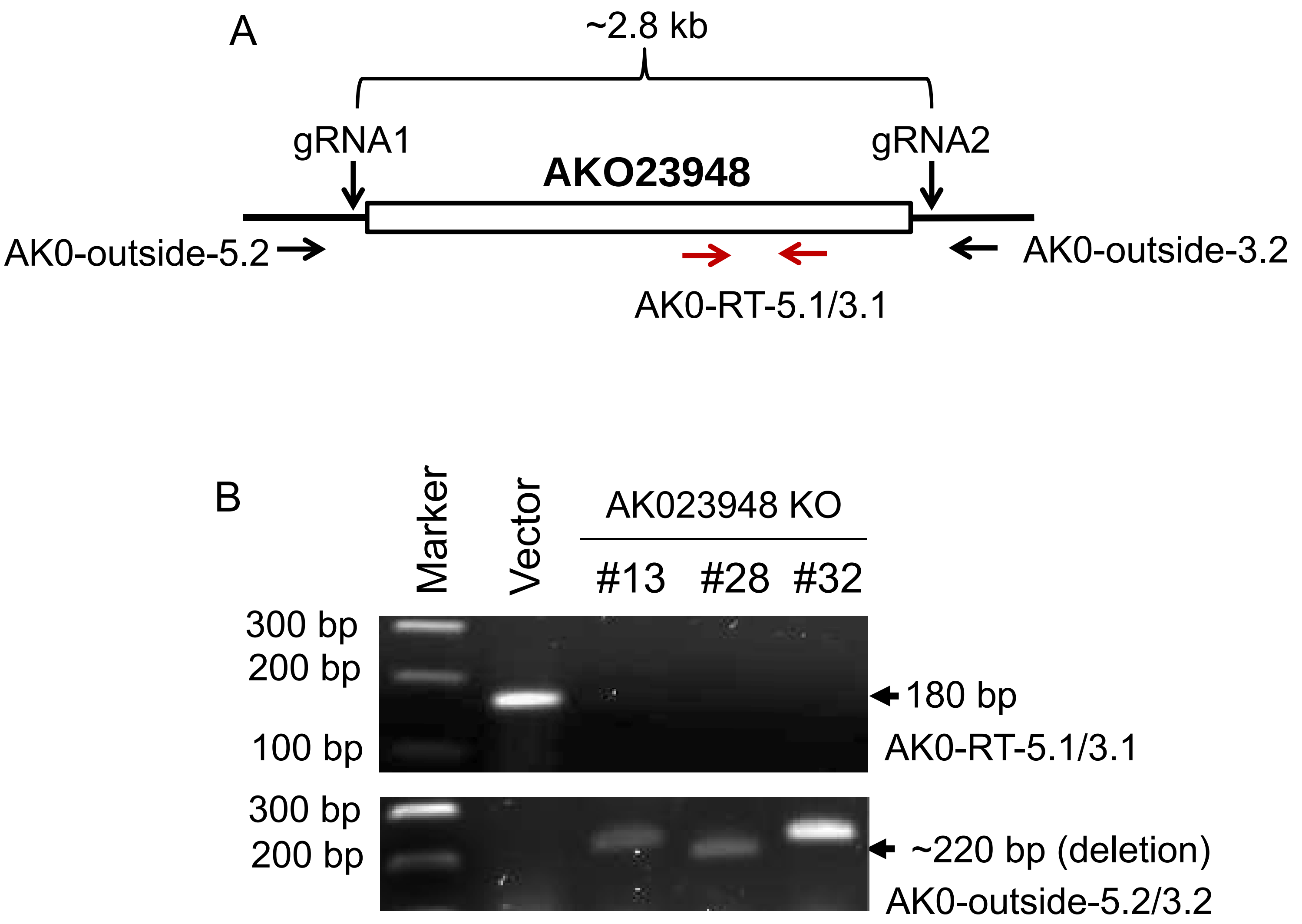
A
~2.8 kb
gRNA1
gRNA2
AKO23948
AK0-outside-5.2
AK0-outside-3.2
AK0-RT-5.1/3.1
B
Marker
Vector
AK023948 KO
#13
#28
#32
300 bp
200 bp
100 bp
180 bp
AK0-RT-5.1/3.1
300 bp
200 bp
~220 bp (deletion)
AK0-outside-5.2/3.2

Fig. S8

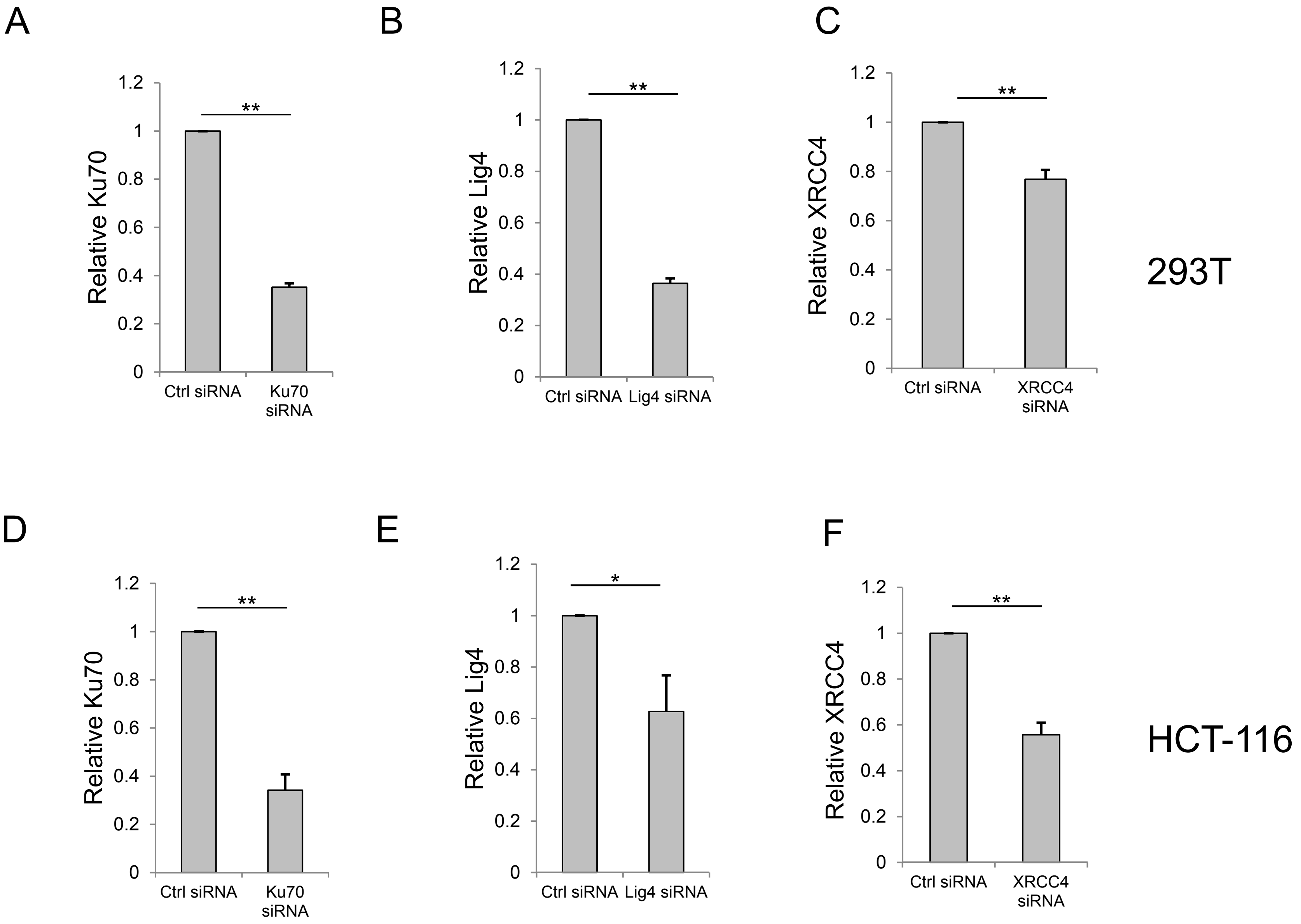
A
B
C
D
E
F
Relative Ku70
Relative Lig4
Relative XRCC4
1.2
1
0.8
0.6
0.4
0.2
0
**
*
Ctrl siRNA
Ku70 siRNA
Lig4 siRNA
XRCC4 siRNA
293T
HCT-116

Fig. S9

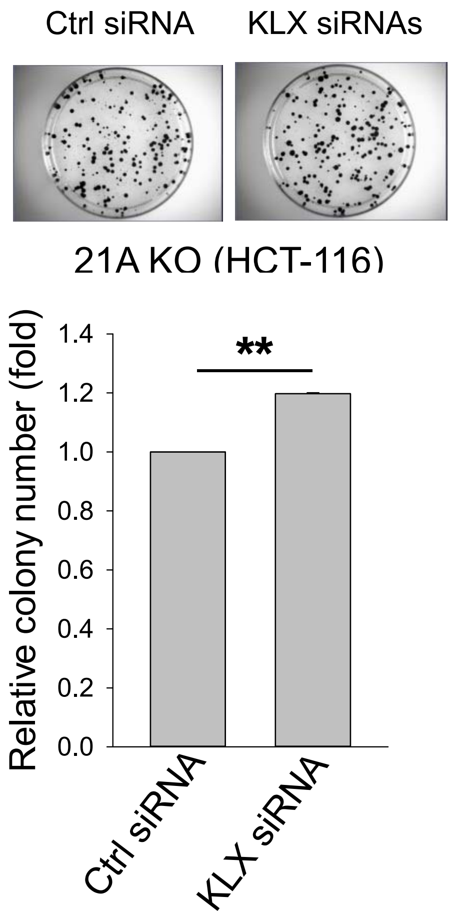

Ctrl siRNA
KLX siRNAs
21A KO (HCT-116)
Relative colony number (fold)
1.4
1.2
1.0
0.8
0.6
0.4
0.2
0.0
**
Ctrl siRNA
KLX siRNA

Fig. S10

Targeting miR-21, UCA1 and lncRNA-21A

Gene	HR event*	
	Vector + donor	gRNAs + donor
miR-21	0/3 (0%)	14/19 (74%)
UCA1	0/3 (0%)	17/20 (85%)
LncRNA-21A	(0%)**	7/8 (87.5%)

* Based on junction PCR products for individual clones. PCR primers are miR-21-recom-5.2/EF1-Seq-3.2 for miR-21, UCA1-recom-5.1/EF1-Seq-3.2, and 21A-recom-5.1A/EF1-Seq-3.2 for lncRNA-21A, respectively.

** A mixed pool of over 10 clones was used for junction PCR.

Supplementary figure legend

Fig. S1 Identification of alterations in targeted region of miR-21 as revealed by DNA sequencing. The sequence on top indicates pre-miR-21 and mature miR-21 sequence is underlined. The sequence carrying the loxP copy is not listed here. Nucleotides in red are insertions and low case cross-outs are deletions. Schematic descriptions are shown on the right.

Fig. S2 Knockout of miR-21 in HCT-116 cells. Knockout of miR-21, as a mixed pool, resulted in high colony number after puromycin selection compared to vector control**.** (A) representative images showing colony formation. (B) Overall results. Values are means of ± SE (n = 3), **, p <0.01. (C) DNA sequences for PCR products from clone #2 and #17. Insertion sequences are shown in blue and cross-cut indicates a deletion. (D) Knockout of miR-21 causes cell growth inhibition as shown by colony formation for clone #2 and #17 compared to vector control.

Fig. S3 Characterization of miR-21 knockout clones in HCT-116 cells. (A) Identification of HR events by junction PCR using primers, as indicated on top panel and junction PCR products at bottom panel. See Fig. 1C for description of miR-21 donor vector. While donor + vector control revealed no HR event from 3 clones, donor + miR-21 gRNAs resulted in ~74% HR rate (14/19). (B) DNA sequencing of three randomly selected PCR products (clone #1, #3 and #14) directly isolated from the gel revealed identical sequences.

Fig. S4 miR-21 knockout in LNCaP (A & B) and MCF-7 cells (C & D), as measured by colony formation after puromycin selection and relative miR-21 expression for a mixed pool as detected by qRT-PCR. Values are means of ± SE (n = 3). *, p <0.05. **, p <0.01.

Fig. S5 UCA1 knockout using a single gRNA or dual gRNA approach. (A) Location of UCA1 gRNAs targeting exon 1 for single gRNA and the gRNA sequence is shown on top with PAM in red. (B) Location of primers used for detection of KO clones by genomic PCR. (C) An identical sequencing result for junction PCR products from clone #3, #5 an d#18 (Fig. 5D).

Fig. S6 Knockout of lncRNA-21A in HCT-116 cells. (A) Strategy for targeting lncRNA-21A, indicating gRNA sites. (B) Colony formation after puromycin selection as a mixed pool. Values are means of ± SE (n = 3). **, p <0.01. (C & D) Identification of lncRNA-21 KO clones by genomic PCR and expected sizes of PCR products. PCR primers OF/OR were able to detect wild type fragment

(640 bp) and deleted fragment (169 bp); junction PCR primers (21A-recom-5.1A/EF1-Seq-3.2) were able to detect HR events (~800 bp). All three clones, but not vector control, carried a deleted fragment and a HR fragment which was verified by DNA sequencing. However, clone S7 still carried a wild type fragment like vector control. Therefore, clones M2 and S38 are complete knockouts, whereas clone S7 is a partial knockout. (E) DNA sequencing of junction PCR products (clone S7, M2 and S38) directly recovered from the gel revealed identical sequences.

Fig. S7 Knockout of AK023948 in MCF-7 cells. (A) Strategy for targeting AK023948, indicating gRNA sites and primers for PCR. (B) Identification of AK023948 KO clones by genomic PCR and relative locations of inside or outside primers. All of three clones are complete knockouts because they all lacked a 180 bp band.

Fig. S8 Suppression of Ku70, Lig4 and XCCR4 by RNAi. (A) Detection of Ku70 (A), Lig4 (B) and XCCR4 (C) in HEK293T cells by qRT-PCR. Detection of Ku70 (D), Lig4 (E) and XCCR4 (F) in HCT-116 cells by qRT-PCR.

Fig. S9 Enhancing the CRISPR/Cas9-mediated targeting efficiency for lncRNA-21A by siRNAs against Ku70, Lig4 and XRCC4 (KLX siRNAs) in HCT-116 cells.

Fig. S10 Detection of HR by junction PCR for miR-21, UCA1 and lncRNA-21A knockout.
